# Inflammation primes the murine kidney for recovery by activating AZIN1 adenosine-to-inosine editing

**DOI:** 10.1172/JCI180117

**Published:** 2024-09-03

**Authors:** Segewkal Hawaze Heruye, Jered Myslinski, Chao Zeng, Amy Zollman, Shinichi Makino, Azuma Nanamatsu, Quoseena Mir, Sarath Chandra Janga, Emma H. Doud, Michael T. Eadon, Bernhard Maier, Michiaki Hamada, Tuan M. Tran, Pierre C. Dagher, Takashi Hato

**Affiliations:** 1 Department of Medicine, Indiana University School of Medicine, Indianapolis, Indiana, USA.; 2 Faculty of Science and Engineering, Waseda University, Tokyo, Japan.; 3 Luddy School of Informatics, Computing, and Engineering, Indiana University, Indianapolis, Indiana, USA.; 4 Department of Biochemistry and Molecular Biology, Indiana University School of Medicine, Indianapolis, Indiana, USA.; 5 AIST–Waseda University Computational Bio Big-Data Open Innovation Laboratory, National Institute of Advanced Industrial Science and Technology, Tokyo, Japan.; 6 Graduate School of Medicine, Nippon Medical School, Tokyo, Japan.; 7 Richard L. Roudebush Veterans Affairs Medical Center, Indianapolis, Indiana, USA.; 8 Department of Medical and Molecular Genetics, Indiana University School of Medicine, Indianapolis, Indiana, USA.

**Keywords:** Nephrology, Bioinformatics, Cell stress, Polyamines

## Abstract

The progression of kidney disease varies among individuals, but a general methodology to quantify disease timelines is lacking. Particularly challenging is the task of determining the potential for recovery from acute kidney injury following various insults. Here, we report that quantitation of post-transcriptional adenosine-to-inosine (A-to-I) RNA editing offers a distinct genome-wide signature, enabling the delineation of disease trajectories in the kidney. A well-defined murine model of endotoxemia permitted the identification of the origin and extent of A-to-I editing, along with temporally discrete signatures of double-stranded RNA stress and adenosine deaminase isoform switching. We found that A-to-I editing of antizyme inhibitor 1 (AZIN1), a positive regulator of polyamine biosynthesis, serves as a particularly useful temporal landmark during endotoxemia. Our data indicate that AZIN1 A-to-I editing, triggered by preceding inflammation, primes the kidney and activates endogenous recovery mechanisms. By comparing genetically modified human cell lines and mice locked in either A-to-I–edited or uneditable states, we uncovered that AZIN1 A-to-I editing not only enhances polyamine biosynthesis but also engages glycolysis and nicotinamide biosynthesis to drive the recovery phenotype. Our findings implicate that quantifying AZIN1 A-to-I editing could potentially identify individuals who have transitioned to an endogenous recovery phase. This phase would reflect their past inflammation and indicate their potential for future recovery.

## Abstract 2

Adenosine-to-inosine editing of antizyme inhibitor 1 (AZIN1) promotes recovery from acute kidney injury by enhancing metabolic flexibility.

## Graphical abstract

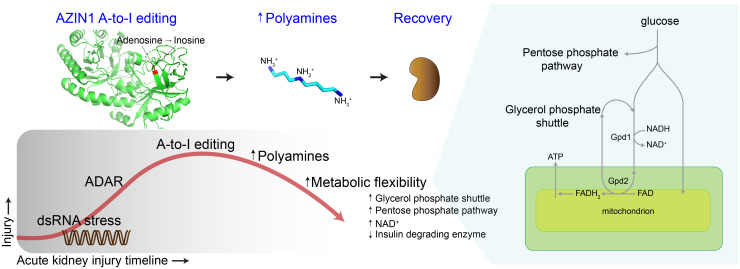

## Introduction

The polyamines — namely putrescine, spermidine, and spermine — are involved in a variety of fundamental biological processes, such as transcription, translation, cell growth, differentiation, DNA repair, and aging (1–3). Polyamines are fully protonated at physiological pH, and a substantial fraction of polyamines are associated with ribosomes (~15%) and RNA (~80%) (4). These nucleotide-bound polyamines facilitate global protein synthesis through their direct interaction with the translation machinery (5, 6). The critical role of polyamines in protein synthesis is further supported by the fact that cancer cells frequently exploit the polyamine pathway to enhance their growth (7). Conversely, polyamines are also essential for the activation of immune cells (8, 9), blurring the boundaries between therapeutic advantages and disadvantages in a variety of settings.

The regulation of polyamine bioavailability is determined by a multitude of mechanisms, including gut absorption, de novo synthesis, and the salvage pathways. In addition, polyamines significantly influence their own pathway through various post-transcriptional mechanisms (1). These mechanisms include ribosomal frameshifting (ornithine decarboxylase antizyme 1), ribosomal occupancy of upstream open reading frames (spermine synthase and spermidine/spermine *N*^1^-acetyltransferase 1), stop codon readthrough (adenosylmethionine decarboxylase 1), and posttranslational modification of eukaryotic translation initiation factor 5A (hypusination), as well as post-transcriptional mRNA editing of antizyme inhibitor 1 (*AZIN1*) from adenosine to inosine (A-to-I). This A-to-I editing results in a non-synonymous amino acid mutation, as inosines are translated as guanosines (10). The presence of these intricate regulatory mechanisms within this pathway underscores the crucial importance of controlling polyamine levels in response to various environmental stresses.

The kidney is an organ with exceptionally high metabolic demands (11), making it susceptible to various stressors such as diabetes and sepsis, which can disrupt polyamine homeostasis. Indeed, a recent study has highlighted that altered polyamine metabolism is a unifying feature across more than 10 different kidney injury models in mice, as well as in the post–kidney transplantation context in humans (12, 13). Although the importance of polyamines in kidney biology is indisputable, their exact role under stress conditions remains unclear. The supplementation of polyamines and the modulation of the polyamine pathway have yielded diverse outcomes in multiple models of kidney injury, ranging from providing protection to exacerbating tissue damage (14–21). These varying results underscore the need for a more systematic examination of the roles of polyamines across specific disease timelines and trajectories.

Defining timelines and stages of any kidney disease is highly challenging. Because of variations in disease progression among patients, a uniform physical timescale cannot be universally applied. We reasoned that the precisely controlled, stepwise reactions embedded in the polyamine pathway could serve as the basis for constructing a molecular clock. This path of investigation has led to our present findings, which demonstrate that *AZIN1* A-to-I editing is strikingly prevalent and occurs at specific points along disease timelines in both mouse models and humans. As such, *AZIN1* A-to-I editing can serve as a molecular clock to stage various forms of kidney disease.

AZIN1 is a key regulatory enzyme that controls the initial entry point into the polyamine pathway by augmenting the activity of ornithine decarboxylase 1 (22). The A-to-I editing of *AZIN1* confers a gain-of-function phenotype, thereby further increasing polyamine biosynthesis. Such gain-of-function *AZIN1* A-to-I editing has been described in several forms of cancer, contributing to aggressive tumor behavior (23–26). The role of *AZIN1* editing is also implicated in hematopoietic stem cell differentiation (27). More recently, transient *AZIN1* editing has been reported in cases of COVID-19 infection (28). However, the clinical implications of *AZIN1* editing in non-cancerous kidney diseases remain unclear.

By combining a series of sequencing and genetic approaches, we found that *AZIN1* edited state confers an advantage over the unedited state by upregulating the polyamine pathway and co-opting glycolysis and nicotinamide biosynthesis, culminating in a metabolically robust phenotype. Using an extensively characterized murine model of endotoxemia, we also provide a genome-wide, time-anchored map of A-to-I editing, serving as a novel framework for the development of molecular staging in kidney disease.

## Results

### AZIN1 A-to-I editing is widespread in non-cancerous conditions.

**Figure 1:**
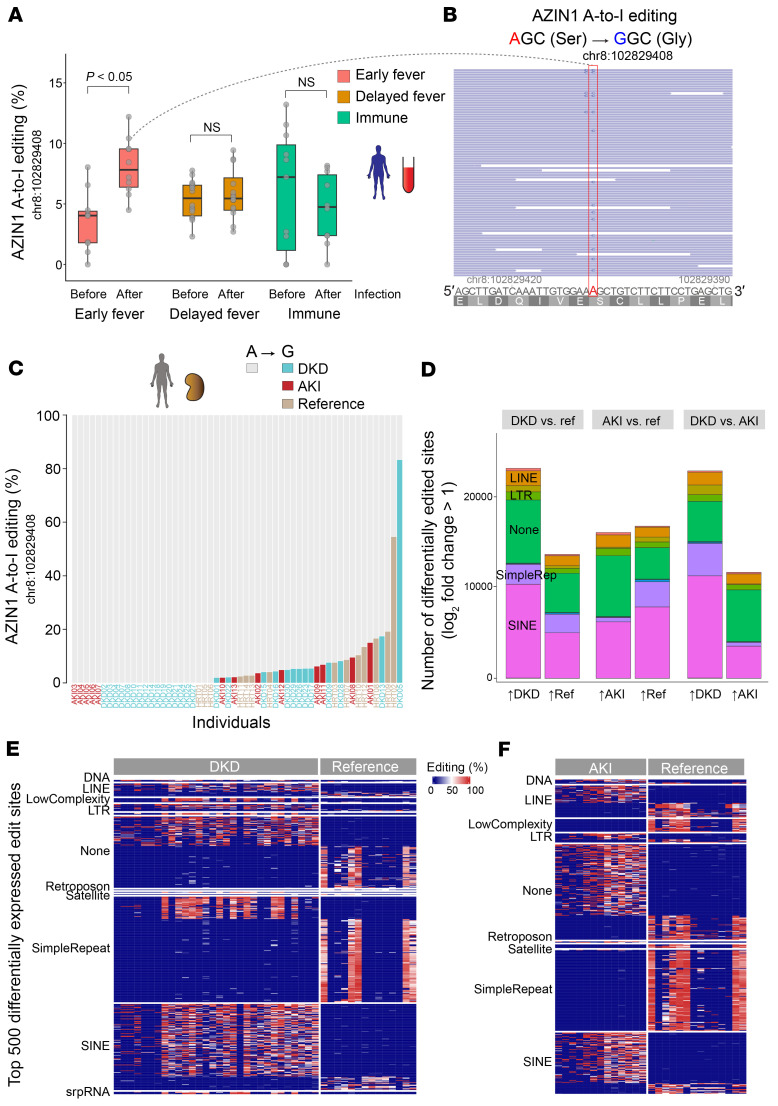
AZIN1 A-to-I editing status in non-cancerous diseases in humans. (**A**) Distribution of *AZIN1* A-to-I editing rates (percent of edited reads over total reads) in prospectively collected blood from male children aged 6–11 years, before and after *Plasmodium falciparum* malaria infection. Individuals were classified as early fever (symptomatic and first-time infection), delayed fever (asymptomatic and first-time infection, subsequently developing malarial symptoms), and immune (infected but never developing symptoms). (**B**) Representative read coverage near the *AZIN1* editing site for one sample. Note that inosine is sequenced as guanosine. The human *AZIN1* gene is encoded on the minus strand, hence the T-to-C mutation, not A-to-G, in the coverage track. Light-blue-colored reads (F2R1 paired-end orientation) indicate the proper directionality of reads mapped to the minus strand. (**C**) Distribution of *AZIN1* A-to-I editing rates in kidney biopsies with a pathology diagnosis of diabetic kidney disease (DKD), acute kidney injury (AKI), or reference nephrectomy samples. Each column represents one sample. (**D**) Stacked bar chart summarizing total numbers of differentially expressed A-to-I editing sites genome-wide under the indicated conditions. For each comparison, editing sites are divided on the *x* axis based on the direction of fold change. For example, in the DKD versus reference comparison, approximately 20,000 sites are more edited in DKD, whereas approximately 10,000 sites are more edited in reference nephrectomy samples. (**E**) Heatmap displaying the top 500 differentially expressed A-to-I editing sites between diabetic nephropathy and reference nephrectomy samples. The differentially expressed sites are categorized based on repeat classes. (**F**) Comparison between AKI biopsies and reference nephrectomy samples.

Using a model of endotoxin preconditioning, we have previously identified that increased polyamine levels are a key feature of the robust protective phenotype against severe sepsis (14). Increases in polyamine levels are also reported by others during the recovery phase of ischemia/reperfusion injury (29). Conversely, inhibiting a branch of the polyamine pathway can also lead to tissue protection against multiple models of kidney diseases (e.g., inhibition of ornithine decarboxylase or eukaryotic translation initiation factor 5A hypusination) (15, 16, 30–32). These contrasting findings suggest that the role of polyamines is context dependent, such as the severity of tissue injury or timing of intervention. To understand the role of polyamines broadly in various stress conditions, here we first interrogated a large clinical data set in which stranded RNA sequencing (RNA-Seq) was performed on whole blood collected from children before and after they contracted malaria (33). Through prospective surveillance, the patients were categorized into (a) early fever (infection with concurrent fever), (b) delayed fever (infection with a delay of 2–14 days until development of fever), and (c) immune (infection without progression to fever). We found that *AZIN1* A-to-I editing at chromosome 8:102829408 (hg38), a known A-to-I editing site (34), was highly prevalent in this cohort, albeit at different time points among the 3 groups (Figure 1, A and B, and Supplemental Figure 1, A–C; supplemental material available online with this article; https://doi.org/10.1172/JCI180117DS1). Notably, children in the early fever group had low levels of *AZIN1* A-to-I editing at baseline but showed an increase in editing after malaria infection. In contrast, children in the delayed and immune groups exhibited surprisingly high levels of A-to-I editing at baseline that were sustained over time. This raises the possibility that *AZIN1* A-to-I editing early in the course of malaria infection could have a beneficial role in controlling disease progression.

Next, we interrogated stranded RNA-Seq data of human kidney biopsies obtained from our biobank and the Kidney Precision Medicine Project (35, 36). We found that *AZIN1* editing is common in non-cancerous kidney tissues, including those with diabetic kidney disease, acute kidney injury (AKI), and even reference nephrectomy (Figure 1C). However, no difference was found in the extent of *AZIN1* editing among the 3 groups. This may be due to the fact that these biopsies were obtained at various stages in the diabetes and AKI timelines (Supplemental Figure 2, A–G; https://connect.posit.iu.edu/bulk_kidney_bx/). Similarly, the reference biopsies are known to sustain variable degrees of ischemic injury, thus exhibiting some AKI phenotype. In addition, some reference nephrectomy samples were derived from tissues adjacent to renal cell carcinoma, which may also influence *AZIN1* A-to-I editing status. Nevertheless, genome-wide examination did reveal significant differences among the 3 groups in A-to-I editing at tens of thousands of sites (Figure 1D; see Methods). Overall, diabetic kidneys showed more extensive genome-wide A-to-I editing than nephrectomies and AKI samples. Focusing on the top differentially edited sites, reference nephrectomy samples had A-to-I editing predominantly within simple repeat regions, whereas AKI and diabetic samples had A-to-I editing within short interspersed nuclear elements (SINEs, such as Alu elements; Figure 1, E and F). The differential editing in transposable elements such as SINEs may have profound implications for disease unfolding (37). No significant A-to-I editing was identified in mitochondrial transcripts for all conditions, implicating no breach of mitochondrial RNA into the cytoplasm (Supplemental Figure 2H) (38).

### Changes in AZIN1 A-to-I editing and polyamine metabolism across AKI timelines.

**Figure 2:**
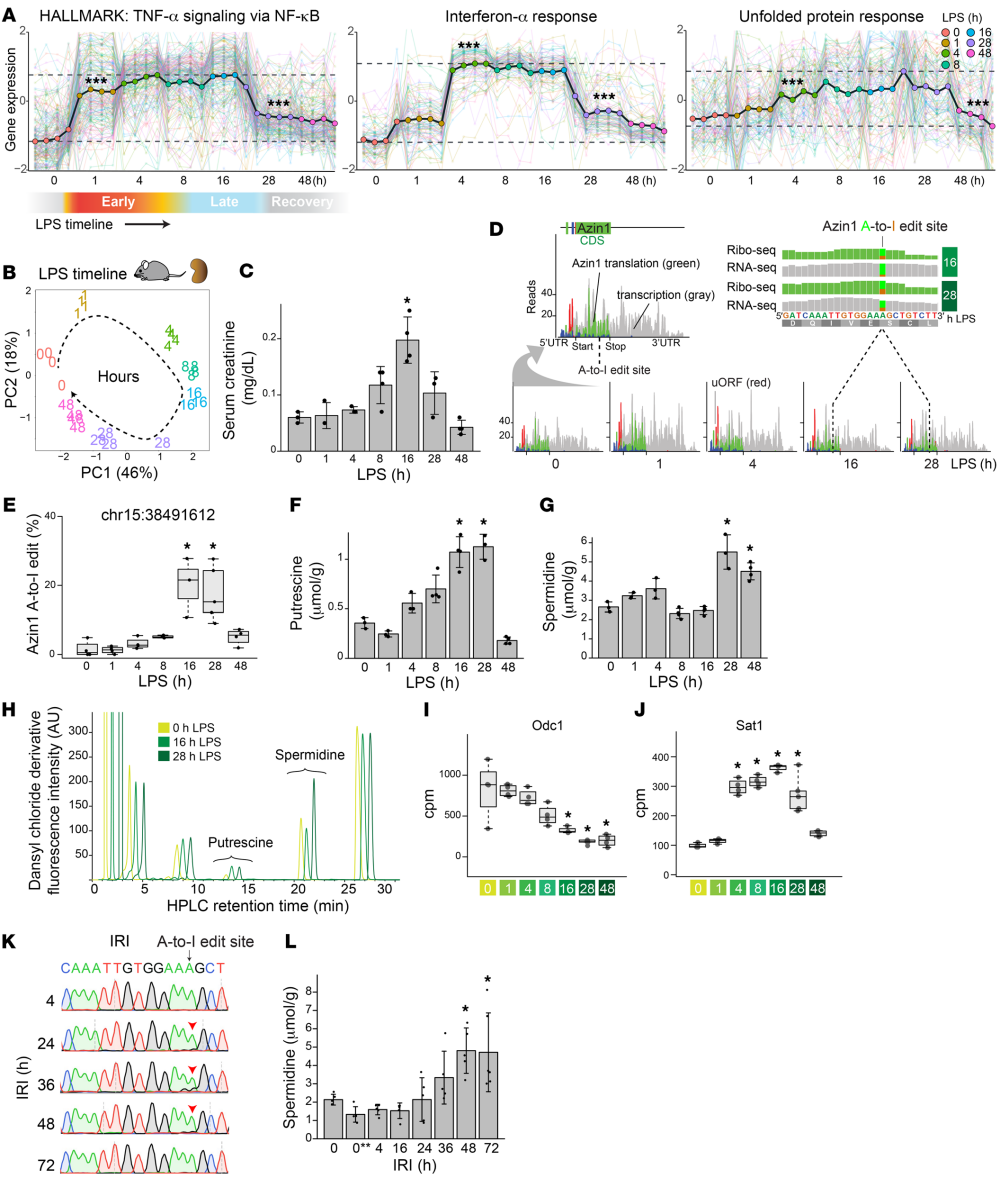
Azin1 A-to-I editing status in murine models of AKI. (**A**) Bulk RNA-Seq analysis on a murine model of endotoxemia (LPS). Gene set coregulation analysis showing sequential upregulation of pathways involved in NF-κB–mediated acute inflammation and in antiviral/interferon responses, followed by the integrated stress response, as indicated by enrichment of the Molecular Signatures Database Hallmark Gene Sets. Each dot corresponds to each animal. The colored lines in the background depict scaled expression of individual genes. ***Pairwise *t* test adjusted *P* < 0.05 compared with the preceding time point. (**B**) Principal component analysis showing overall gene expression changes over the course of endotoxemia in the kidney. (**C**) Serum creatinine levels at indicated time points after administration of LPS (4 mg/kg in C57BL/6J male mice). (**D**) Combined Ribo-Seq and RNA-Seq read coverage graphs for *Azin1* after LPS challenge in the kidney. Reads are mapped to Ensembl transcript *Azin1*-201. Gray-colored reads represent RNA-Seq, whereas red/green/blue-colored reads represent codon frames for ribosome-protected fragments in Ribo-Seq. The top right panel confirms the translation of A-to-I–edited *Azin1* (reanalysis of GEO GSE120877). (**E**) Percentage of Azin1 A-to-I editing under indicated conditions (based on stranded total RNA-Seq data). (**F**–**H**) Measurements of kidney tissue putrescine and spermidine levels by HPLC under indicated conditions. Representative HPLC chromatograms are also shown. For clarity, the traces are slightly shifted from each other on the *x* axis elution time. (**I** and **J**) Quantitation of RNA-Seq read counts (in counts per million) at the indicated time points. (**K**) Sanger sequencing showing timeline-specific *Azin1* A-to-I editing observed in wild-type mouse kidneys after ischemia/reperfusion injury (IRI; arrowheads). (**L**) Measurements of kidney tissue spermidine levels by HPLC after IRI. *P < 0.05 vs. 0-hour control samples, 1-way ANOVA followed by Dunnett’s test for multiple treatment comparisons. 0** indicates kidney tissues harvested 20 minutes after ischemia without reperfusion.

To understand the role of *AZIN1* editing and polyamine metabolism in the kidney, we next interrogated a well-characterized animal model of endotoxemia (39–41). In this specific model, the kidney goes through precise stages, starting with classic NF-κB–mediated acute inflammation, followed by interferon responses and the integrated stress response, and culminating in metabolic and translation shutdown (Figure 2, A–C). Single-cell RNA-Seq revealed that *Azin1* is expressed in all cell types in the kidney (Supplemental Figure 3A). Furthermore, Ribo-Seq analysis (ribosome profiling) showed that *Azin1* translation remained nearly constant throughout the course of endotoxemia (Figure 2D). However, we found that *Azin1* A-to-I editing status varied significantly over the same time period (Figure 2E and Supplemental Figure 3B). While the extent of A-to-I editing was minimal at baseline and during the early phases of endotoxemia, it significantly increased during the later stages of sepsis in this model. In fact, we observed a consistent and robust increase in *Azin1* A-to-I editing at around 16 hours and later time points after endotoxin exposure. We have previously shown that this 16-hour time point corresponds to a critical transition phase between translation shutdown and subsequent tissue recovery (39, 40). Thus, editing of *Azin1* at this precise time point may serve as a clock to stage endotoxemia. Furthermore, since *AZIN1* A-to-I editing confers a gain of function (23–26), it may also signal a change in polyamine metabolism that aids tissue healing.

Indeed, quantitation of polyamines in kidney tissues revealed a notable increase in spermidine levels during the recovery phase of endotoxemia (Figure 2, F–H). This increase was observed despite a significant decrease in the expression of ornithine decarboxylase 1, the rate-limiting step of polyamine biosynthesis, and an increase in spermidine/spermine *N*^1^-acetyltransferase 1, the main polyamine catabolic enzyme (Figure 2, I and J, and Supplemental Figure 3C). These findings suggest that the gain-of-function *Azin1* A-to-I editing plays a crucial role in limiting polyamine depletion at the peak of injury and expediting the restoration of tissue polyamine levels during recovery. Single-cell RNA-Seq data implicate that the source of polyamines could be cell type specific, with arginine serving as the substrate for myeloid cells, S3 proximal tubule, and the thick ascending loop of Henle, while proline serves as the substrate for other tubular segments (Supplemental Figure 3, D and E).

Finally, using a murine model of renal ischemia/reperfusion injury, we further extended our analysis of *Azin1* A-to-I editing and polyamine levels. We observed overlapping editing kinetics and polyamine trajectories over the course of ischemic kidney injury compared with endotoxemia. However, the exact timelines differed between the 2 models, and the peak of *Azin1* A-to-I editing and polyamine rebound were delayed after ischemia/reperfusion injury (Figure 2, K and L, and Supplemental Figure 4, A–F).

### AZIN1 A-to-I–uneditable cells are compromised upon nutrient deprivation and mitochondrial inhibition.

**Figure 3:**
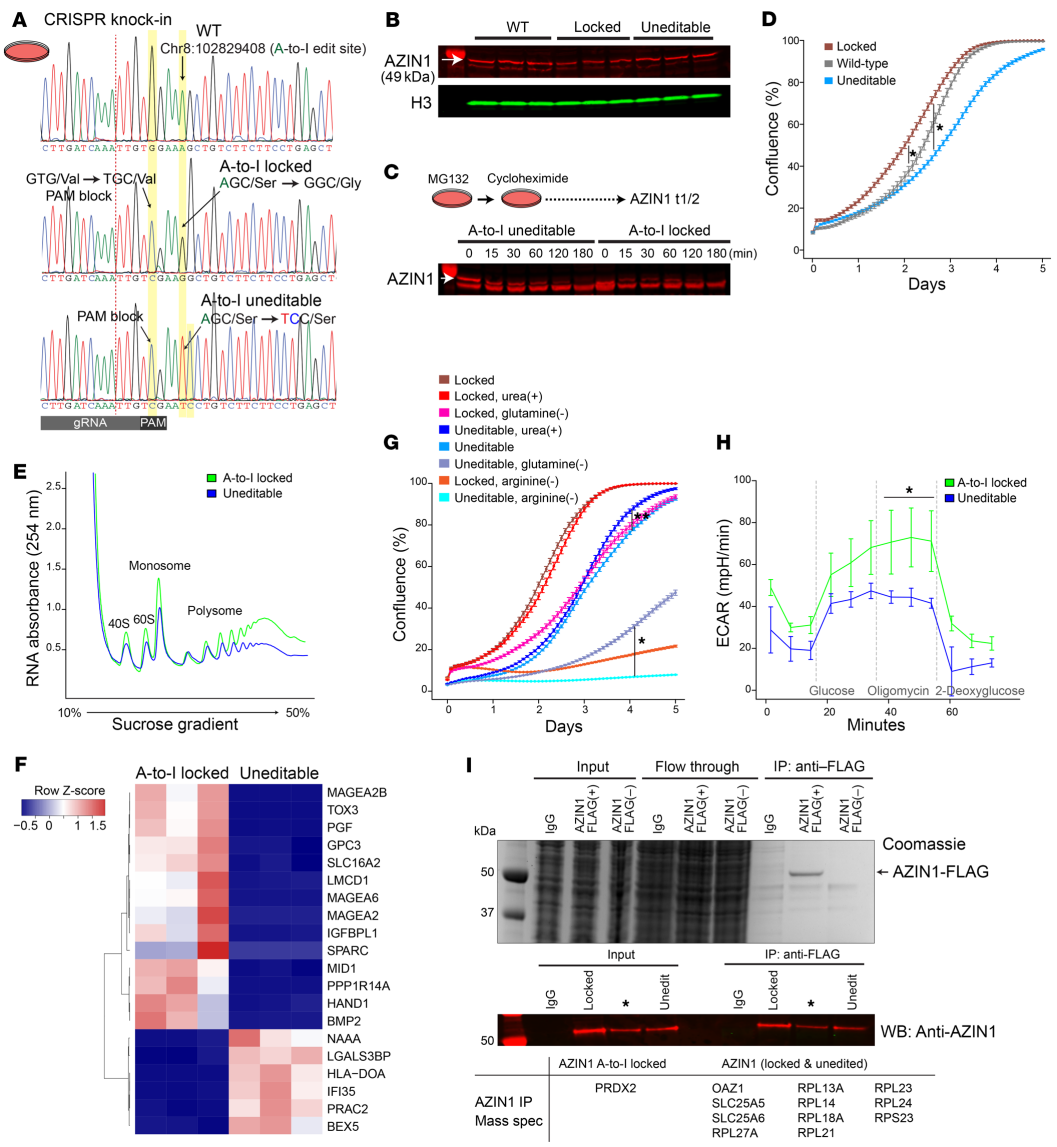
Azin1 A-to-I–uneditable state hinders cell growth and limits glycolytic capacity. (**A**) Sanger sequencing chromatograms for wild-type (HEK293T; top), *AZIN1* A-to-I–locked (middle), and *AZIN1* A-to-I–uneditable homozygous cell lines (bottom). Homology-directed repair donor oligonucleotides used for CRISPR knockin are shown in Supplemental Figure 5A. (**B**) Western blotting for AZIN1 under indicated conditions (~70% confluence). (**C**) Determination of AZIN1 protein turnover under indicated conditions. Nascent protein synthesis was inhibited with 250 μg/mL cycloheximide. Arrow points to AZIN1. Bands below AZIN1 result from inhibition of proteasomal degradation with MG132. *n* = 2 biological replicates. (**D**) Real-time monitoring of cell growth for AZIN1 A-to-I–locked, uneditable, and wild-type cells. *n* = 3 independent experiments with *n* = 6 technical replicates for each experiment. *P < 0.05 at all time points for indicated conditions, except the stationary phase between AZIN1 A-to-I–locked and wild-type cells. Representative images are shown in Supplemental Figure 5C. (**E**) Polyribosome profiling of AZIN1 A-to-I–locked and uneditable cell lines. *n* = 3 independent experiments. Mean polysome/monosome ratios for A-to-I–locked and uneditable genotypes are 4.1 and 3.6, respectively. (**F**) Heatmap of the top 20 differentially expressed genes between AZIN1 A-to-I–locked and uneditable cell lines as determined by RNA-Seq (https://connect.posit.iu.edu/azin1/). (**G**) Cell growth under indicated conditions. Representative images are shown in Supplemental Figure 5D. *P < 0.05, **P < 0.05 after day 1 and day 2.5 for indicated conditions, respectively. (**H**) Extracellular acidification rates under indicated conditions (Seahorse glycolysis stress test). *n* = 3 independent experiments with *n* = 3 technical replicates for each experiment. *P < 0.05 vs. AZIN1-uneditable cells at indicated time points. (**I**) Identification of AZIN1-interacting molecules by mass spectrometry. Top: Coomassie staining for input, flow-through, and immunoprecipitated unfractionated lysates from IgG control and transfection of FLAG-tagged AZIN1 or AZIN1 without FLAG plasmids. Middle: Western blotting for AZIN1. Cells overexpressing FLAG-tagged A-to-I–locked AZIN1 or uneditable plasmids were fractionated into cytoplasmic and nuclear compartments and immunoprecipitated using anti-FLAG antibody (cytoplasmic fraction is shown). See also Supplemental Figure 5H. Summary of coprecipitated proteins with AZIN1 is presented in the bottom table. *n* = 3 independent experiments. *Plasmid construct not used in this article.

To elucidate the functional significance of *AZIN1* editing, we next designed 2 homozygous clonal cell lines using the CRISPR knockin strategy (Figure 3A and Supplemental Figure 5A). The first cell line contains a constitutively edited *AZIN1*, resulting in an A-to-I–locked state (AGC serine to GGC glycine). The second cell line is an A-to-I–uneditable variant in which the editing site is disrupted while preserving the codon composition (AGC serine to TCC serine). A-to-I–locked or uneditable state did not lead to changes in the abundance or stability of the AZIN1 protein (Figure 3, B and C). We found that A-to-I–locked cells exhibited accelerated cell growth compared with wild-type and A-to-I–uneditable cells, all of which share an otherwise identical genetic background (HEK293T; Figure 3, D and E, and Supplemental Figure 5, B and C). The level of A-to-I editing in the wild-type cells was minimal (~0%). However, the growth curve of the wild-type cells fell between those of the A-to-I–locked and uneditable cells. This suggests that transient and low-grade *AZIN1* editing is operative under normal conditions, contributing to healthy cellular growth. In support of the rapid growth rate observed in the A-to-I–edited state, multiple genes involved in cell growth and differentiation were upregulated in the A-to-I–locked cell line (e.g., *BMP2*/bone morphogenetic protein 2, *IGFBPL1*/insulin-like growth factor–binding protein like 1, *PGF*/placental growth factor; Figure 3F and Supplemental Figure 5E; https://connect.posit.iu.edu/azin1/).

As expected, the depletion of arginine exhibited a profound growth-inhibitory effect on cell proliferation, which was more notable in the uneditable cell line (Figure 3G and Supplemental Figure 5D). Conversely, the supplementation of urea, known to enhance polyamine biosynthesis (42, 43), rescued cell proliferation in the uneditable cell line. This effect was not observed in the A-to-I–locked cell line, suggesting that polyamine synthesis is maximized in the A-to-I–locked state. In addition, the impact of glutamine depletion was less pronounced in the A-to-I–locked cell line (Figure 3G). Surprisingly, glycolysis stress test revealed marked differences in extracellular acidification rates between A-to-I–locked and uneditable cell lines. Specifically, the uneditable cell line lacked a compensatory glycolytic response upon ATP synthase inhibition (Figure 3H and Supplemental Figure 5, F and G). While the exact mechanism remains uncertain, these findings offer a new perspective on the involvement of *AZIN1* A-to-I editing in metabolic flexibility. This is especially pertinent in situations such as cancer and ischemia/reperfusion injury. In this regard, non-polyamine-related functions of A-to-I–locked AZIN1 cannot be excluded. For example, immunoprecipitation of AZIN1 identified that A-to-I–edited AZIN1 uniquely binds to the thiol-specific peroxidase peroxiredoxin 2 (Figure 3I and Supplemental Figure 5H).

### Azin1 A-to-I editing confers resilience through the orchestration of multiple protective pathways.

**Figure 4:**
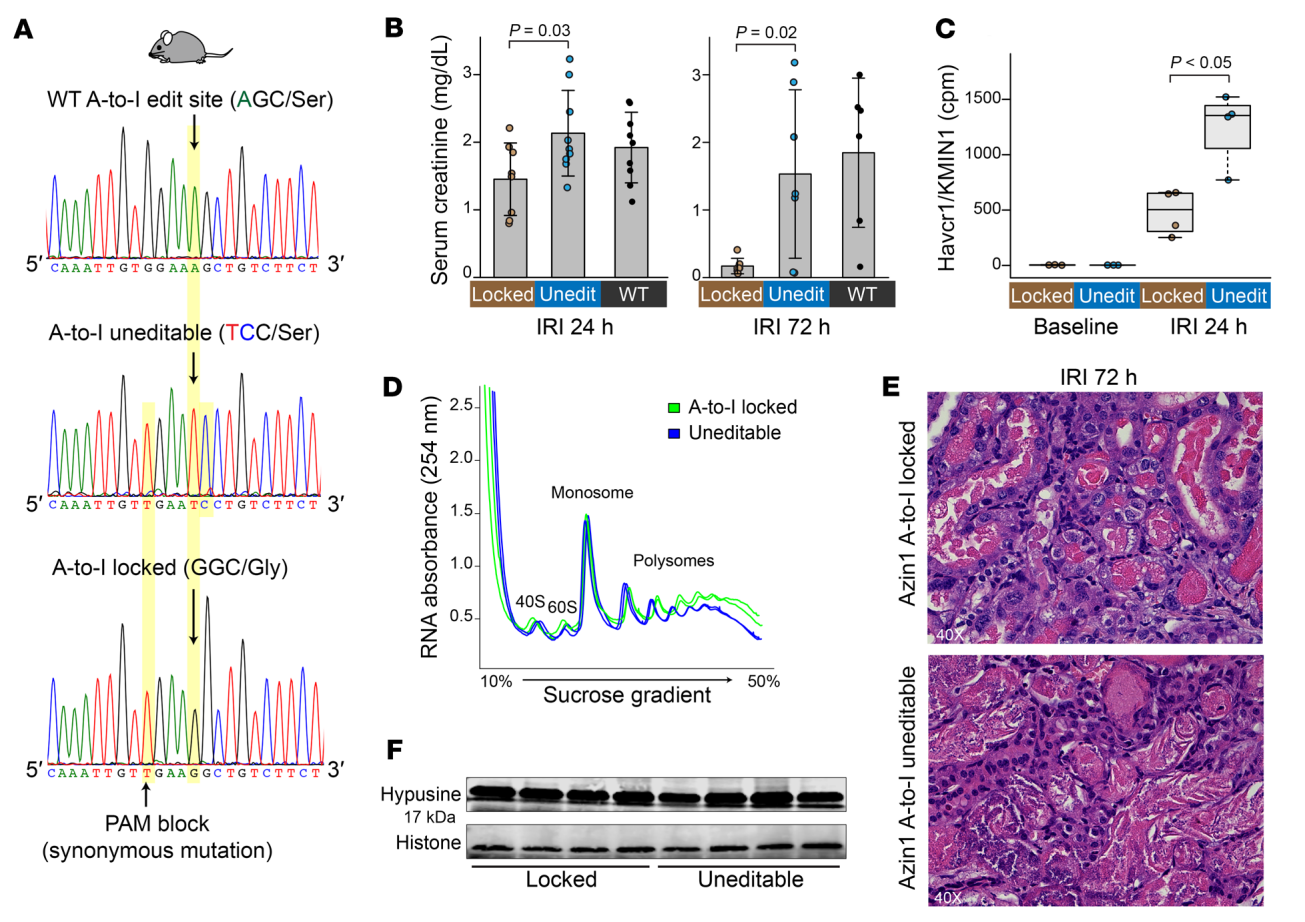
Azin1 A-to-I–locked mice exhibit faster tissue recovery following ischemic injury compared with uneditable mice. (**A**) Sanger sequencing chromatograms for wild-type (top), Azin1 A-to-I–uneditable (middle), and Azin1 A-to-I–locked homozygous mice (bottom). The CRISPR knockin strategy is depicted in Supplemental Figure 6A. (**B**) Serum creatinine levels 24 and 72 hours after a 20-minute bilateral IRI. (**C**) Kidney tissue Havcr1/kidney injury marker-1 (KIM1) levels as determined by RNA-Seq (counts per million). (**D**) Polyribosome profiling of kidneys from Azin1 A-to-I–locked and uneditable mice 24 hours after IRI. Two representative biological replicates are shown for each genotype. Mean polysome/monosome ratios for A-to-I–locked and uneditable genotypes are 3.3 and 2.8, respectively. (**E**) Hematoxylin and eosin staining 72 hours after IRI. Original magnification, ×40. (**F**) Western blotting for hypusine in the kidney after IRI.

To gain further insight into the in vivo implications of *Azin1* A-to-I editing, we next created 2 CRISPR knockin mouse models, representing both A-to-I–locked and uneditable states (Figure 4A and Supplemental Figure 6A). Mutant mice were born at the expected Mendelian ratios with no gross abnormalities (Supplemental Figure 6, B–F). Because Azin1 edited status had a significant effect on glycolysis, we examined its role in an ischemia/reperfusion model of kidney injury. We found that Azin1-locked mice had less severe kidney damage after ischemia/reperfusion injury as compared with the uneditable mice (Figure 4, B and C). In addition to the reduction in serum creatinine and tissue *Havcr1*/KIM1 levels, the less pronounced tissue damage in A-to-I–locked mice was reflected in better-preserved global translation and a faster resolution of tubular necrosis (Figure 4, D and E, and Supplemental Figure 7A). Note that no discernible difference was observed in the hypusination of eukaryotic translation initiation factor 5A between the 2 knockin models (Figure 4F). This suggests that the beneficial effects of *Azin1* A-to-I editing on translation are mediated through hypusination-independent polyamine pathways.

**Figure 5:**
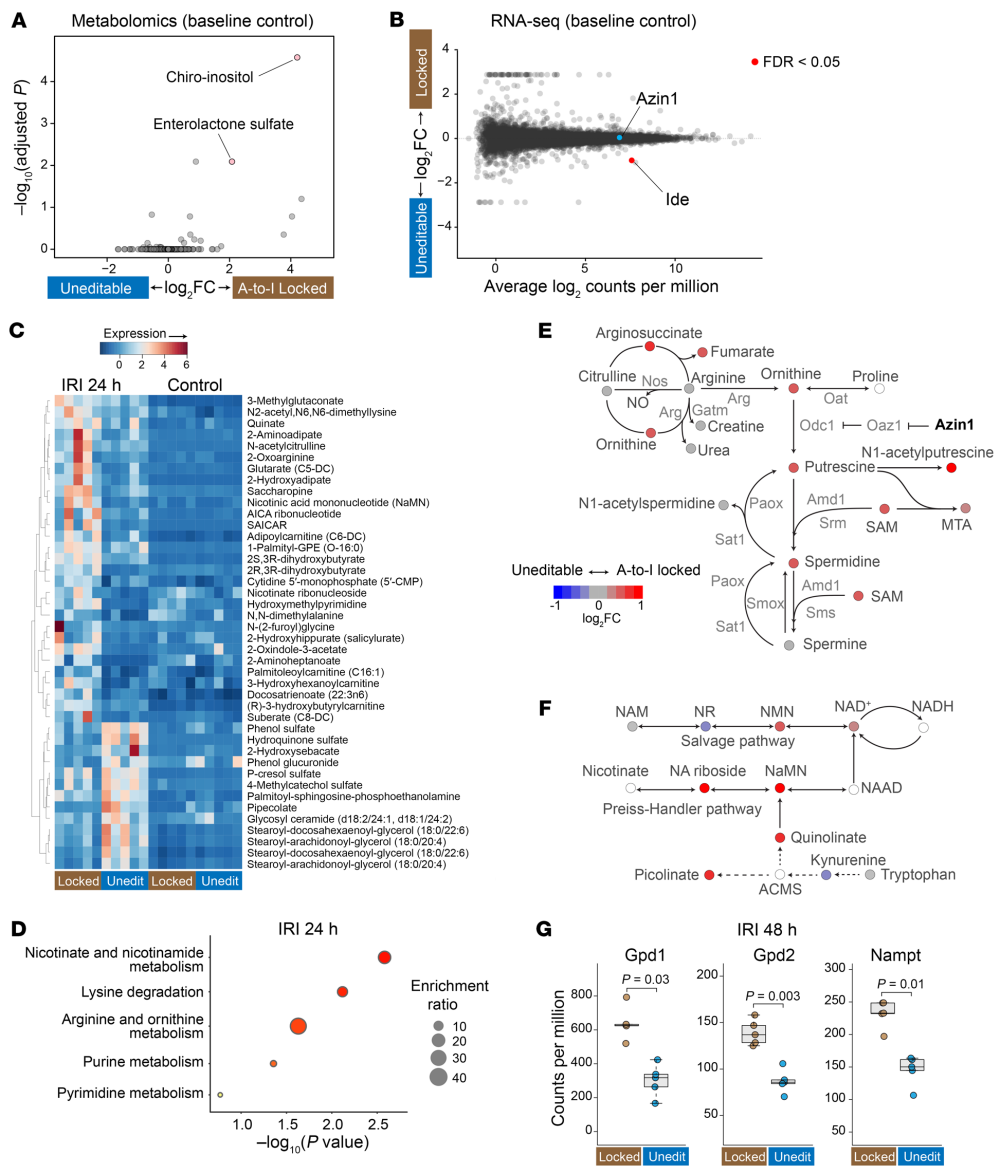
Azin1 A-to-I–locked state limits kidney injury by upregulating polyamines and other protective pathways. (**A**) Volcano plot showing the top 2 differentially expressed metabolites. The *x* axis depicts the log_2_ fold change of A-to-I locked/uneditable ratio, and the *y* axis depicts –log_10_ adjusted *P* values. Global untargeted metabolomics, *n* = 5 for each condition. (**B**) RNA-Seq gene expression analysis (smear plot) comparing homozygous A-to-I–locked and uneditable mouse kidneys under basal conditions. Only Ide (insulin-degrading enzyme) met the criteria of FDR < 0.05 (https://connect.posit.iu.edu/azin1_mouse_kidney/). (**C**) Heatmap displaying the top differentially expressed metabolites between Azin1-locked and uneditable mice after IRI (adjusted *P* < 0.05 for all listed metabolites). (**D**) Pathway enrichment analysis of differentially expressed metabolites between Azin1 A-to-I–locked and uneditable mouse kidneys after IRI. (**E**) Metabolite ratios (log_2_ fold change of A-to-I locked/uneditable) mapped to the polyamine pathway and pseudocolored according to the indicated scale. Metabolites with blank circles were not resolved by the metabolomics. (**F**) Metabolite ratios mapped to the NAD^+^ biosynthesis pathway. (**G**) RNA-Seq read counts for glycerol-3-phosphate dehydrogenase 1, cytoplasmic (Gpd1), glycerol-3-phosphate dehydrogenase 2, mitochondrial (Gpd2), and nicotinamide phosphoribosyltransferase (Nampt), 48 hours after IRI.

Metabolomics analysis yielded surprisingly few differentially expressed metabolites at baseline in these 2 mouse models (Figure 5A). Specifically, only enterolactone sulfate and chiro-inositol were elevated in the kidneys of A-to-I–locked mice compared with A-to-I–uneditable mice. While the function of enterolactone sulfate remains unclear (weakly estrogenic; ref. 44), chiro-inositol is a well-characterized metabolite known to facilitate the conversion of pyruvate to acetyl-CoA through the dephosphorylation of pyruvate dehydrogenase (45, 46). The endogenous synthesis of chiro-inositol is catalyzed by insulin-dependent epimerases (47), and the dephosphorylation of pyruvate dehydrogenase is central to providing metabolic flexibility (48). Remarkably, RNA-Seq revealed insulin-degrading enzyme (*Ide*) (49, 50) as the sole differentially expressed gene in these 2 mouse models under basal conditions (Figure 5B and Supplemental Figure 8, A and B; https://connect.posit.iu.edu/azin1_mouse_kidney/). The expression of insulin-degrading enzyme was significantly downregulated in A-to-I–locked mice, promoting insulin signaling in the A-to-I–locked state. Thus, the heightened chiro-inositol level, facilitated by the downregulation of insulin-degrading enzyme, could explain the resilience of the A-to-I–locked state against mitochondrial insults such as ischemia/reperfusion injury and direct ATP synthase inhibition as shown above (Figure 3H). An inverse correlation was also observed in the human reference kidney biopsies between the degree of *AZIN1* A-to-I editing and the levels of insulin-degrading enzyme (Supplemental Figure 8C). Insulin-degrading enzyme is also known to degrade amyloid β (50, 51). No amyloid β deposits were observed in our mouse models (Supplemental Figure 8D).

In contrast to basal conditions, we identified multiple differentially expressed metabolites following ischemia/reperfusion injury in these 2 mouse strains (24 hours after ischemia; Figure 5, C and D, and Supplemental Figure 8E). First, A-to-I–locked state resulted in global upregulation of metabolites involved in the polyamine pathway, including *S*-adenosylmethionine, which serves as a donor of amine groups essential for the synthesis of higher-order polyamines (spermidine and spermine; Figure 5E). Using HPLC and tissue staining, we confirmed that higher levels of polyamines were sustained in the A-to-I–locked state during the recovery phase of ischemic injury (48–72 hours after ischemia; Supplemental Figure 9, A and B). Interestingly, A-to-I–locked mice showed increased NAD^+^ levels following ischemia/reperfusion injury (Figure 5F). The beneficial effects of NAD^+^ have been extensively characterized across various animal models and human studies (52). We also found that A-to-I–locked mice had higher levels of AICAR (5-aminomidazole-4-carboxamide ribonucleotide), which originates from the pentose phosphate shunt/purine metabolism (Figure 5D and Supplemental Figure 8E). The elevated AICAR levels under ischemic stress could result from the augmented glycolytic capacity conferred by the A-to-I–locked condition. AICAR operates as a potent endogenous AMPK activator, contributing to a multitude of cellular protection mechanisms (53). Notably, RNA-Seq analysis revealed that the 2 most significantly increased transcripts in the A-to-I–locked state at 48 hours after ischemia were (a) nicotinamide phosphoribosyltransferase (*Nampt*), an enzyme involved in NAD^+^ salvage, and (b) glycerol-3-phosphate dehydrogenase 2 (*Gpd2*), the mitochondrial glycerophosphate dehydrogenase involved in the glycerol phosphate shuttle. This shuttle system produces ATP via FADH_2_ in the mitochondria and regenerates NAD^+^ in the cytoplasm (Figure 5G and Supplemental Figure 10, A–D). In addition, glycerol-3-phosphate dehydrogenase 1 (*Gpd1*), the cytosolic counterpart required for coupling of the shuttle system, was among the top 20 transcripts significantly upregulated in the A-to-I–locked state. Altogether, our findings indicate that *Azin1* A-to-I editing renders cells resilient to ischemic stress by harnessing multiple protective pathways. These pathways encompass the upregulation of polyamine biosynthesis, NAD^+^ biosynthesis, glycerol phosphate shuttle, and pentose phosphate shunt/purine metabolism. Finally, we examined the role of *Azin1* A-to-I editing in the endotoxemia model and confirmed the renoprotective effects of A-to-I–locked state (Supplemental Figure 10E).

### Origin of dsRNA species.

**Figure 6:**
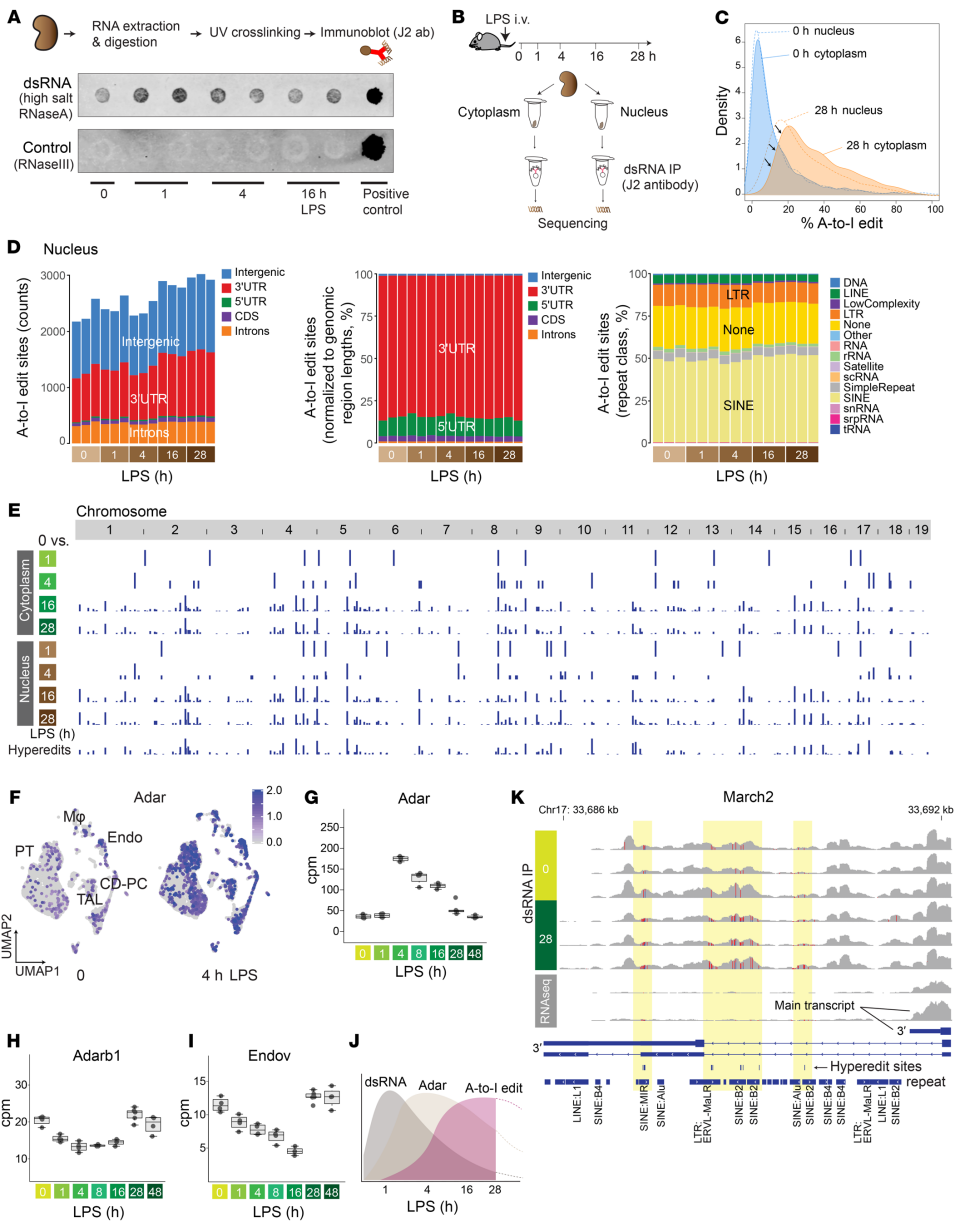
Genome-wide characterization of A-to-I editing in mouse kidneys. (**A**) Immunoblotting of dsRNA under indicated conditions. RNase A incubation was done with high salt to specifically digest single-stranded RNA. The negative control consisted of RNase III digestion, which digests dsRNA. The positive control consisted of poly(I:C) without RNase digestion. (**B**) Schematic representation of dsRNA immunoprecipitation and sequencing. (**C**) Overlay of density plots displaying A-to-I edit percentages under indicated conditions. (**D**) Left: Total counts and distribution of A-to-I editing sites per sample (nuclear fraction; editing rate > 10% and reads count > 5 in at least 3 samples; see Methods for further pre-processing criteria). Middle: Distribution of A-to-I editing sites, normalized to genomic region lengths. Right: Distribution of A-to-I editing sites per repeat class. CDS, coding sequence. (**E**) Summary of A-to-I editing sites that exhibit differential expression compared with the 0-hour baseline. The bottom track represents hyper-editing sites. (**F**) Single-cell uniform manifold approximation and projection (UMAP) displaying the distribution of *Adar* expression in the mouse kidney (reanalysis of published data GEO GSE151658). PT, proximal tubule; CD-PC, collecting duct principal cell; TAL, thick ascending loop of Henle. (**G**–**I**) Quantitation of total RNA-Seq read counts (in counts per million) at the specified time points. (**J**) Scheme depicting the sequence of events observed in the kidney. (**K**) Read coverage comparison for *March2* near the transcription termination site between dsRNA enrichment (top 6 tracks) and without dsRNA enrichment (bottom 2 tracks, 0 and 28 hours after LPS; regular total RNA sequencing).

A-to-I editing is catalyzed by the enzyme adenosine deaminase, RNA specific (ADAR), which specifically binds to double-stranded RNA (dsRNA) structures (54). To investigate the nature of dsRNA species involved, we examined kidneys from our murine model of endotoxemia. Immunoblotting revealed an acute increase in dsRNA levels 1 hour after endotoxin challenge (Figure 6A). dsRNA may arise from repetitive elements resembling virus-like structures, such as long terminal repeats (LTRs) and non-LTR retrotransposons (SINEs and LINEs) (55). PCR analysis of select repeat elements revealed an increase of MusD (type D murine LTR retrotransposons) 4 hours after endotoxin challenge in the kidney (Supplemental Figure 11A). However, in general, our select PCR targets did not show consistent results, suggesting that the origin of dsRNA may not be repeat class specific.

To further investigate the phenomenon of dsRNA stress (54), we next conducted immunoprecipitation of dsRNA species, followed by stranded RNA-Seq on cytoplasmic and nuclear fractions (dsRNA-Seq; Figure 6B and Supplemental Figure 11, B–F). The dsRNA-specific monoclonal antibody (J2) effectively enriched transcripts of varying lengths, ranging from approximately 40 base pairs to several thousand base pairs (Supplemental Figure 11D). This is in line with the antibody’s known characteristics (56). In the early course of endotoxemia, the abundance of dsRNA transcripts mapping to gene body regions correlated well with those from conventional total RNA-Seq, suggesting that the initial surge of dsRNA burden primarily consists of acute inflammatory molecules (Supplemental Figure 12, A–C). In comparison with conventional total RNA-Seq, dsRNA-Seq enriched mitochondrially encoded RNA transcripts at baseline and during the early stages of endotoxemia (Supplemental Figure 11E). This observation is consistent with the fact that (a) mitochondrial transcription is highly active in the kidney at early time points (Supplemental Figure 12D; mitochondrial transcription decreases at later time points), and (b) mitochondrial transcripts are prone to forming dsRNA structures owing to the bidirectional transcription of the mitochondrial genome (Supplemental Figure 12E) (38). Immunoprecipitation of dsRNA species also led to the enrichment of intergenic transcripts (Supplemental Figure 11E). A more detailed examination revealed that these intergenic dsRNAs were particularly common in regions adjacent to the gene coding regions (±10 kb from transcription start and end sites; Supplemental Figure 12F). These regions are prone to various mechanisms that can induce the generation of antisense reads, thereby facilitating dsRNA formation (57, 58).

In addition to antisense reads, intramolecular base pairing of single-stranded RNA (stem-loop) is another important source of dsRNA structures that can be catalyzed by ADAR. As demonstrated in Supplemental Figure 12G, sufficiently long complementary repeat regions are present in many genes, especially within intronic regions (912 genes for 30 bp cutoff). These repeat regions contribute to the enrichment of various gene body regions, including introns within our dsRNA-Seq data set.

### Characterization of A-to-I editing sites.

Having identified dsRNA species that could be targets of ADAR, we next examined the broad distribution of A-to-I editing sites in the mouse kidney. Our data revealed millions of A-to-I editing sites distributed across the genome (see Methods). However, the majority of these editing sites had low coverage (≤5 reads), minimal editing levels (a few percent), or inconsistent editing patterns per condition. Thus, we implemented stringent filtering criteria and focused our analysis on approximately 3,000 editing sites of high confidence for the rest of this study. Importantly, our analytical pipeline employed sequential alignment procedures (Supplemental Figure 11B), enabling the capture of hyper-editing sites that will otherwise fail to map to a reference genome because of an excess of mismatches (59).

Across the genome, we found that both the extent of editing per site and the number of edited sites increased during the later stages of endotoxemia (Figure 6, C–E, and Supplemental Figure 13, A–C). A comparison of the cytoplasmic and nuclear fractions revealed that A-to-I editing occurred predominantly in the nucleus. Nevertheless, although the nucleus remained the primary site of editing, a greater number of transcripts underwent editing within the cytoplasm during the late phase of endotoxemia (Figure 6C). This transition was preceded by an upregulation of *Adar* expression across all cell types in the kidney (Figure 6, F–H; the paralog of *Adar*, *Adarb1*, was downregulated). In parallel, the expression of endonuclease V, the inosine-specific endoribonuclease (60), decreased, which would also contribute to the preservation of A-to-I–edited transcripts (Figure 6I).

In summary, our comprehensive time-course analysis delineated the sequence of events leading to A-to-I editing: initiation with dsRNA stress (1 hour), followed by *Adar* overexpression (4 hours), and culminating in an increase in A-to-I editing (16 hours) (Figure 6J). The same sequence of events was also observed after ischemia/reperfusion injury (Supplemental Figure 14, A–C). Representative dsRNA-Seq read coverage tracks for each endotoxemia time point are available on a genome browser at https://connect.posit.iu.edu/view_GY/

**Figure 7:**
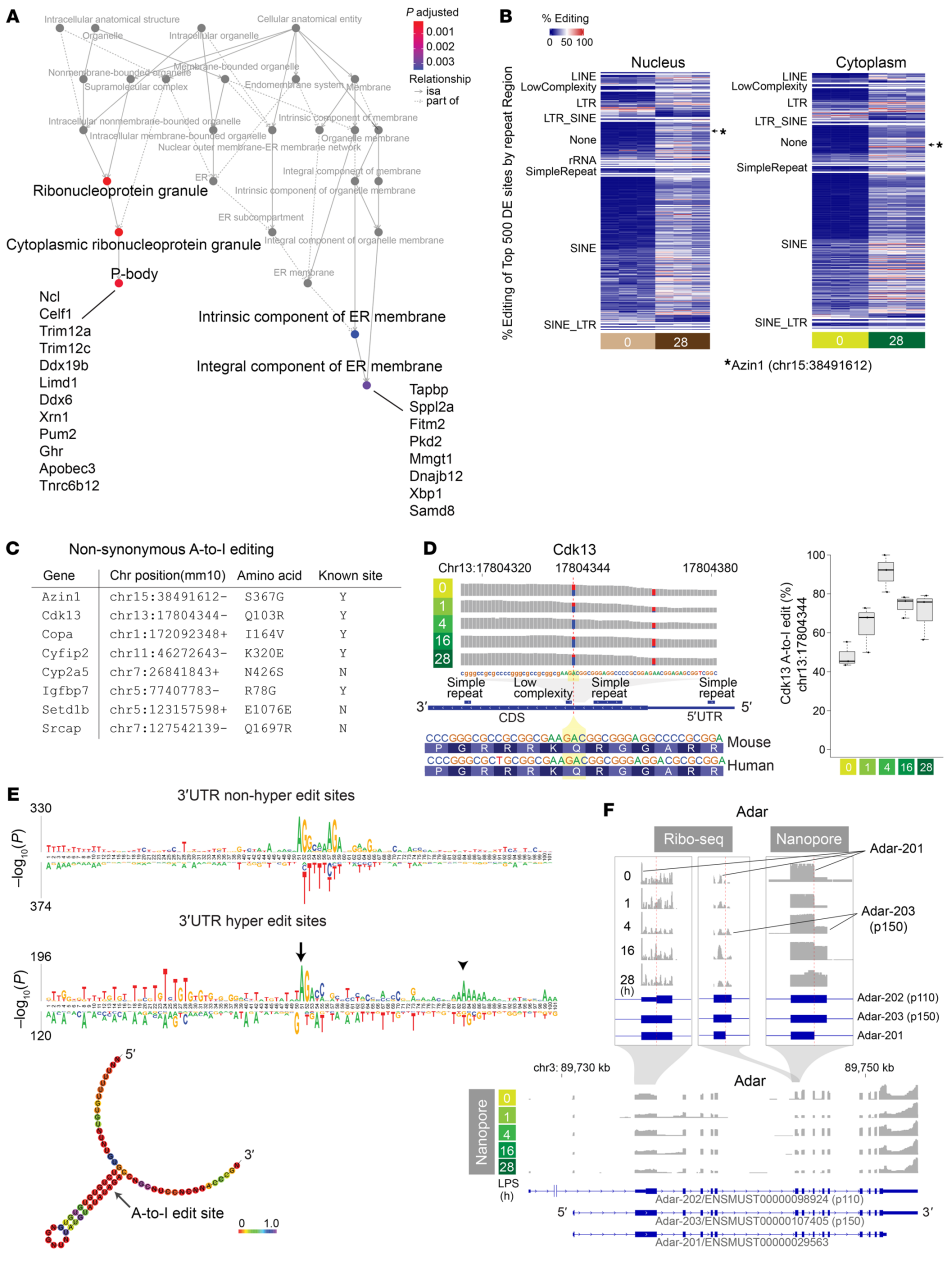
Genome-wide characterization of A-to-I editing in mouse kidneys. (**A**) Pathway enrichment analysis based on genes that exhibit differential editing rates between baseline and 28 hours (cytoplasmic compartment). isa, inferred from sequence alignment. (**B**) Heatmap displaying the top 500 differentially expressed A-to-I editing sites between 0-hour baseline and 28 hours after endotoxin in the kidney. The differentially expressed (DE) sites are categorized based on repeat classes. (**C**) List of genes exhibiting non-synonymous A-to-I coding sequence mutation in response to an endotoxin challenge in the kidney. (**D**) *Cdk13* reads distribution and A-to-I editing under indicated conditions. (**E**) Comparison of motif enrichment between non-hyper-editing (top) and hyper-editing sites (bottom) within ±50 nucleotides centered around A-to-I editing sites. Predicted RNA secondary structure around the 3′-UTR hyper-editing site is shown at the bottom (arrow). Positional entropy is color-coded. (**F**) Ribo-Seq and nanopore read coverage graphs for *Adar*, clarifying *Adar* transcript isoform switches during endotoxemia.

Over the entire endotoxemia time course, A-to-I editing was most prominent in 3′-untranslated regions (3′-UTRs) (Figure 6D and Supplemental Figure 13A). This prevalence of A-to-I editing in the 3′-UTR was also pronounced in hyper-editing sites (Supplemental Figure 14, D–F). As expected, editing occurred preferentially in repeat regions, especially in SINEs (Figure 6D, Supplemental Figure 13A, and Supplemental Figure 14, E and F). No significant temporal changes were observed in the overall proportion of edit sites per repeat class. We observed markedly different read coverage distribution between dsRNA-Seq and total RNA-Seq for certain genes. For example, *March2*, an E3 ubiquitin ligase involved in antiviral and antibacterial immune responses, showed significant transcription readthrough with respect to the canonical transcription termination site across a series of hyper-edited regions (Figure 6K). This phenomenon of readthrough was not readily apparent in conventional RNA-Seq data, suggesting that these heavily edited transcripts might be lowly expressed or unstable (Supplemental Figure 15, A and B). The enrichment of dsRNA reads in intronic regions, specifically in repeat regions, was also notable (Supplemental Figure 15, C and D). The high prevalence of A-to-I editing in the intronic regions indicates that editing takes place immediately on nascent transcripts prior to splicing. Given that A-to-I editing in intronic regions could potentially impact alternative splicing (61), we further scrutinized individual editing sites. Nearly all edit sites were found outside of splicing donor or acceptor regions, including the branch point adenosine (Supplemental Figure 15E). Pathway enrichment analysis revealed that differentially edited sites are enriched in genes related to the regulation of ribonucleoproteins/P-bodies and the unfolded protein response/endoplasmic reticulum membrane (Figure 7, A and B).

### A-to-I editing within coding sequence regions.

A-to-I editing within coding sequences was exceedingly rare. Specifically, instances of A-to-I editing that led to non-synonymous mutations were identified only in the following genes: *Azin1*, *Cdk13*, *Copa*, *Cyfip2*, *Cyp2a5*, *Igfbp7*, *Setd1b*, and *Srcap* (Figure 7C). Cdk13 functions as a transcriptional cyclin-dependent kinase involved in nuclear RNA surveillance. The A-to-I editing event in the *Cdk13* coding sequence occurred near the N-terminus between 2 repeat regions, resulting in a glutamine-to-arginine mutation (Figure 7D). This particular editing site is conserved across both mice and humans (34). We found that the rate of editing at this site was markedly elevated at baseline and increased even further after endotoxin challenge (Figure 7D). Notably, this editing site was recently linked to aggressive cancer phenotypes (62), analogous to the findings with *Azin1* A-to-I editing.

In the case of *Azin1*, editing at chromosome 15:38491612 (mm10) results in a serine-to-glycine mutation. The rate of *Azin1* editing significantly increased from 0% to over 40% with the progression of endotoxemia (Supplemental Figure 16, A and B). When the serine-to-glycine mutation occurred, the neighboring adenosine was also edited in approximately 50% of cases, resulting in a synonymous mutation (chr15:38491613). Isolated editing of this adjacent adenosine was rare, confirming that chr15:38491612 is indeed the primary editing site. There was a complete absence of A-to-I editing 2 nucleotides away from the main editing locus. These findings underscore the remarkable precision and highly predictable nature of A-to-I editing. Because the *Azin1* editing site is located near the alternative splice site, we also conducted nanopore long-read RNA-Seq and determined that the *Azin1* editing status does not correlate with alternative splicing (Supplemental Figure 16, C and D).

### Adar isoform switching in the mouse kidney.

While the clinical implications of A-to-I editing at individual sites remain largely unknown and some are likely inconsequential, various studies have underscored the significance of A-to-I editing in controlling the kinetics of transcripts, RNA-RNA interactions, R-loop formation, and RNA-protein interactions (63, 64). Generally, A-to-I editing serves to disrupt a long stretch of complementary base pairing, thereby attenuating the binding of dsRNA sensors such as PKR and MDA5 (65). Our motif enrichment analysis revealed ADAR’s preference for editing adenosines adjacent to guanosines (5′AG3′; Figure 7E), consistent with prior reports (66, 67). Intriguingly, when focusing on hyper-editing sites, we detected satellite A-rich regions situated approximately 30 nucleotides downstream of the primary editing site within the 3′-UTR (Figure 7E, bottom track). ADAR has been shown to edit recursively at a fixed interval of approximately 30 bp downstream of an editing site (68).

Using nanopore long-read RNA-Seq and Ribo-Seq, we identified that a relatively less characterized isoform, *Adar*-201/ENSMUST00000029563, predominates in the murine kidney at baseline and up to 4 hours after endotoxin treatment (Figure 7F). The more widely recognized Adar isoforms are p110 (*Adar*-202/ENSMUST00000098924) and p150 (*Adar*-203/ENSMUST00000107405) (69). The constitutively expressed p110 (in other tissues) lacks a nuclear export signal, hence exerting its editing effect almost exclusively within the nucleus. In contrast, the interferon-inducible isoform p150 harbors both nuclear export and nuclear localization signals, enabling its shuttling between the cytoplasm and nucleus. Similarly, the 201 isoform possesses both nuclear export and nuclear localization signals identical to those of p150, permitting *Adar*-201 to distribute in both compartments. However, unlike the p110 and p150 isoforms, exon 7 of *Adar*-201 is truncated by 26 amino acids as a result of alternative splicing. This splicing occurs at the juncture of the critical dsRNA-binding domain R_III_ (70). Therefore, it could potentially disrupt the editing capacity and account for the absence of significant editing by ADAR in murine kidney tissue at baseline.

In summary, our comprehensive analysis of the endotoxemia model provided a timeline-specific landscape of A-to-I editing, represented by an array of previously undescribed and established editing loci. The phenomenon of A-to-I editing is highly reproducible and quantitative, offering potential for the development of more accurate diagnostic and staging strategies for kidney disease.

## Discussion

The fast and variable progression of AKI poses a major challenge in implementing a stage-specific therapy at the bedside. We have previously identified that translation shutdown is a hallmark of late-phase septic AKI (39, 41). While transient inhibition of protein synthesis could be cytoprotective as it attenuates energy consumption and upregulates the integrated stress response, persistent inhibition of protein synthesis is detrimental. Importantly, in a reversible model of AKI, this late phase is also a crucial transition period in which tissue recovery begins (40). How the tissue, under severe stress, reboots and attains a recovery phenotype is unclear.

In this study, we demonstrate that *Azin1* A-to-I editing plays a key role in promoting tissue recovery after AKI. Leading up to this robust *Azin1* editing is a series of stress responses the kidney goes through. These include NF-κB–mediated acute inflammation, interferon responses, and the integrated stress response, all culminating in metabolic shutdown (39–41, 71). Thus, *Azin1* A-to-I editing represents a landmark outcome following prolonged cellular stress. We found that the lack of *AZIN1* editing renders cells susceptible to nutrient deprivation and attenuates glycolytic reserve, thereby restricting cell proliferation. Conversely, *Azin1* A-to-I editing confers better fitness by coupling increased polyamine bioavailability to the activation of cytoprotective molecules such as NAD^+^ and AICAR. The phenotypic impact of *Azin1* A-to-I editing in vivo is subtle under basal conditions but becomes apparent during stress. This indicates that *Azin1* A-to-I editing itself is not a driver of metabolic rewiring but assists this process during emergency. Collectively, these findings suggest a general model in which *Azin1* A-to-I editing serves as a rational autoregulatory system, safeguarding against sustained metabolic shutdown and providing a cue for tissue recovery.

Our study also provides a comprehensive map of A-to-I editing in the kidney using a model of endotoxemia. This model is highly reproducible and has been extensively characterized (14, 39, 40, 72–74). This model was also independently benchmarked by Zhou et al. against a range of kidney injury models (75), further confirming the distinct stage transition from injury to recovery captured by this model. Our genome-wide interrogation of A-to-I editing revealed that A-to-I editing was enriched in genes involved in crucial stress response pathways including P-bodies and the unfolded protein response during the recovery phase of kidney injury (e.g., *Limd1*, *Celf1*, *Pum2*, *Apobec3*, *Sppl2a*, *Dnajb12*, and *Xbp1*). Given the biological relevance, these editing sites might have evolved to diversify their transcript repertoires or to evade the recognition by dsRNA sensors under stress conditions. The latter mechanism has been clearly demonstrated for A-to-I editing in the kidney disease risk gene *APOL1* by Riella et al. (76).

Infections and various environmental factors frequently act as triggers and exacerbate the progression of kidney disease. The resulting outcomes exhibit significant variability. The present study portrays a timeline-specific role for A-to-I editing in the kidney during periods of stress. This structured transcriptional variation is quantitative and tied to an individual’s unique past. While not all the editing sites are necessarily pertinent or carry biological significance, it is our hope that further clarification of these attributes will enhance the accuracy of disease diagnosis and provide a molecular clock to guide therapy.

### Limitations of the study.

The underlying mechanisms involved in the metabolic flexibility conferred by *AZIN1* A-to-I editing require further investigation. Polyamines are involved in a wide variety of cellular processes, such as DNA/RNA stabilization and protein synthesis. The versatile nature of polyamines makes it challenging to pinpoint precisely where and how they induce metabolic reprogramming. Edited *AZIN1* may also have polyamine-independent roles. Additionally, the role of *AZIN1* editing may vary depending on the type of injury (e.g., sterile inflammation vs. viral or bacterial infection) and the affected tissues. Finally, translating this work to human diseases and controlling *AZIN1* A-to-I editing remain challenging. In this regard, the development of clinical trials using ADAR-based RNA editing technology is highly exciting (77, 78).

## Methods

Further information can be found in Supplemental Methods.

### Sex as a biological variable.

Our human study examined male and female subjects, and similar findings are reported for both sexes. However, in the animal study, only male mice were used, owing to known differences in susceptibility to renal ischemia injury. Nevertheless, the findings in mice are expected to be relevant for both sexes.

### Malaria cohort.

RNA-Seq FASTQ files were obtained from Gene Expression Omnibus (GEO) GSE52166 (stranded total RNA-Seq with 2 × 100 bp paired-end configuration). The study details have been outlined previously (33). This longitudinal cohort study consisted of biweekly active malaria surveillance and passive surveillance through self-referral over a 3-year period. RNA-Seq and PCR were conducted on whole blood samples obtained from subjects before and after *Plasmodium falciparum* infection as determined through the prospective surveillance program.

### Human kidney biopsy.

This study complied with all related ethical regulations. Human sample experiments followed relevant guidelines and regulations. Bulk RNA-Seq data files were obtained from 2 sources: the Biopsy Biobank Cohort of Indiana (GSE139061) and the Kidney Precision Medicine Project Atlas (https://atlas.kpmp.org/repository; accessed January 25, 2023) (35, 36, 79, 80). These bulk kidney tissues were processed from an OCT block using the SMARTer Stranded Total RNA-Seq Kit v2 (Takara). Sequencing was performed in a 2 × 75 bp paired-end configuration using a NovaSeq platform (Illumina).

### Generation of AZIN1 A-to-I–locked and A-to-I–uneditable homozygous clonal cell lines.

We designed single-guide RNAs and single-stranded oligo DNA nucleotides (ssODNs; homology-directed repair donor oligonucleotides), and generated knockin HEK293T cell lines using the CRISPR/Cas9 system. A target knockin and protospacer adjacent motif block (PAM synonymous mutation; GTG/valine to TGC/valine) were introduced in the vicinity of the double-strand break (±10 bp) using the asymmetric donor DNA strategy (36 bp | cut | 91 bp for the nontarget strand).

The sgRNA (+PAM) used for both A-to-I–locked and uneditable genome editing was 5′TGATGAGCTTGATCAAATTG(TGG)3′.

The ssODN (antisense strand) for A-to-I–locked state (AGC/serine to GGC/glycine) was 5′GCAGATGGTTCATGGAAAGAATCTGCTCCCATGTTATCAAAGATAAGCCAATCTCCCACATTCAGCTCAGGAAGAAGACAGCc TTCgACAATTTGATCAAGCTCATCACAGGATGGACCCCAAAGGC3′.

The ssODN (antisense strand) for A-to-I–uneditable state (AGC/serine to TCC/serine) was 5′GCAGATGGTTCATGGAAAGAATCTGCTCCCATGTTATCAAAGATAAGCCAATCTCCCACATTCAGCTCAGGAAGAAGACAGga TTCgACAATTTGATCAAGCTCATCACAGGATGGACCCCAAAGGC3′.

Cells were cultured in 10 cm plates to 70% confluence before nucleofection. Approximately 150 × 10^3^ cells (5 μL) were mixed with 1.49 μL of ssODN (100 μM) and Cas9 complex consisting of 18 μL SF 4D-Nucleofector X solution plus supplement 1 (Lonza V4XC-2012), 6 μL of sgRNA (30 pmol/μL), and 1 μL of Cas9 2NLS nuclease, *Streptococcus* *pyogenes* (20 pmol/μL; Synthego). Nucleofection was done using Amaxa 4D-Nucleofector X (CM-130 program, Lonza). Cells were seeded in 15 cm plates at various concentrations. Clonal isolation was done manually. DNA extraction was done using Quick DNA Miniprep kit (Zymo Research D3025). PCR was done using Q5 High-Fidelity DNA polymerase (New England Biolabs) and Monarch PCR Cleanup Kit (New England Biolabs T1030). PCR primers used were: 5′ACTCACAAATTCAATACCTGCGT3′ (forward) and 5′TGCCTTAAAATAAAATCACCTTACCA3′ (reverse).

PCR products were electrophoresed in 2% agarose gel (TopVision Agarose Tablets, Thermo Fisher Scientific R2801), and bands were excised and extracted using QIAQuick Gel Extraction kit (Qiagen 28706). Sanger sequencing was done at GeneWiz. Software used for design and analysis of mutant cell lines included CRISPRdirect, SnapGene, Primer3Plus, New England Biolabs Tm calculator, and Synthego ICE. Successful homozygous mutant cell lines were chosen for downstream experiments.

### Generation of Azin1 A-to-I–locked and A-to-I–uneditable mouse models.

Similar to the human cell lines, we designed the following sgRNA and ssODNs to generate A-to-I–locked and A-to-I–uneditable mouse models.

The sgRNA (+PAM) used for both A-to-I–locked and uneditable genome editing was 5′TGATGAGCTTGATCAAATTG(TGG)3′.

The ssODN (antisense strand) for A-to-I–locked state (AGC/serine to GGC/glycine) was 5′GCAGATGGTTCGTGGAAAGAATCTGCTCCCATGTTATCAAAGATAAGCCAATCTCCCACATTCAGCTCAGGAAGAAGACAGCc TTCaACAATTTGATCAAGCTCATCACAGGATGGACCCCAAAGGC3′.

The ssODN (antisense strand) for A-to-I–uneditable state (AGC/serine to TCC/serine) was 5′GCAGATGGTTCGTGGAAAGAATCTGCTCCCATGTTATCAAAGATAAGCCAATCTCCCACATTCAGCTCAGGAAGAAGACAGga TTCaACAATTTGATCAAGCTCATCACAGGATGGACCCCAAAGGC3′.

Embryo manipulation and generation of founder mice on C57BL/6J background were performed by The Jackson Laboratory Mouse Model Generation Services. N1 sperms (heterozygous) have been cryopreserved at The Jackson Laboratory (stock 414244 for Azin1 A-to-I–locked and stock 413737 for Azin1 A-to-I–uneditable mice). PCR primers used for genotyping were 5′TGAGACTTATGCCTGATCGTTG3′ (forward) and 5′GGTTCGTGGAAAGAATCTGC3′ (reverse). PCR primers used for cDNA Sanger sequencing were 5′ACAAGGAAGATGAGCCTCTG3′ (forward) and 5′AGCTGGCCTCTGAAAATCAT3′ (reverse).

### Animal models of kidney injury.

Azin1 A-to-I–locked and uneditable mice were housed at Indiana University School of Medicine under a 12-hour light/12-hour dark cycle at 25°C. For studies that did not require the knockin mice, C57BL/6J mice were obtained from The Jackson Laboratory. All mice were 8–12 weeks of age and weighed 24–32 g. Animals were subjected to a single-dose, 4 mg/kg endotoxin (LPS) tail vein i.v. injection in a volume of 300 μL (*E*. *coli* serotype 0111:B4, MilliporeSigma). Untreated mice were given an equivalent volume of sterile normal saline as a vehicle. Ischemia/reperfusion injury was performed under isoflurane anesthesia. Before surgery, mice were given extended-release buprenorphine at a dose of 3.25 mg/kg. The mice were subjected to a 20-minute bilateral renal pedicle clamp followed by reperfusion. A heating pad was used to ensure that their rectal temperature remained above 36°C throughout the surgical procedure. No antibiotics or fluid resuscitation were administered.

### Cells.

HEK293T cells, AZIN1 A-to-I–locked cells, and uneditable homozygous clonal cells were cultured in DMEM (4.5 g/L glucose, l-glutamine, and Na pyruvate; Corning 10-013-CV) with 10% FBS (Midwest Scientific USDAFBS) and 100 U/mL penicillin and 100 μg/mL streptomycin (Thermo Fisher Scientific). All cell types were cultured at 37°C with 5% CO_2_. Hypoxia experiments were done after addition of 250 μL of 1 M HEPES buffer to 10 mL of DMEM in each 10 cm dish. After the filling of the hypoxia chamber with nitrogen, cells were incubated for 3 hours. 

### dsRNA immunoprecipitation.

We adopted and made modifications to existing protocols (38, 81). Mouse kidneys were harvested and immediately minced on an ice-cold dish. One-third of the minced tissue was transferred to 1.2 mL of fractionation/lysis buffer, which consisted of 10 mM Tris (pH 7.0), 10 mM NaCl, 5 mM MgCl_2_, 0.5% IGEPAL CA-630 (MilliporeSigma I8896), 0.5% Triton X-100, DNase I (10 U/mL; Zymo E1011A), and Superase-In (2 μL per 1 mL; Thermo Fisher Scientific). The lysate was centrifuged at 3,000*g* for 3 minutes at 4°C. The supernatant was further centrifuged at 21,000*g* for 5 minutes. The resulting supernatant represents the cytoplasmic fraction. The pellet obtained from the initial centrifugation was resuspended in 1 mL of the fractionation/lysis buffer and homogenized using a Minilys tissue homogenizer (Bertin Instruments) at the highest speed for 45 seconds. After homogenization, the lysate was centrifuged at 21,000*g* for 5 minutes. This supernatant serves as the nuclear fraction. Each fraction was then incubated with anti-dsRNA monoclonal antibody J2 (SCICONS/Jena Bioscience RNT-SCI-10010200; IgG2a κ light chain) at a concentration of 10 μg per 600 μL of lysate for 2 hours at 4°C. Mouse IgG2a κ (clone eBM2a; eBioscience 14-4724-82) was used as an isotype control. Protein G Dynabeads (Invitrogen 10003D) were washed in the immunoprecipitation buffer described below and then incubated with the sample-antibody mix for 1 hour at 4°C. The dsRNA-antibody-Dynabeads complex was washed on a magnetic rack using 500 μL washed 4 times with immunoprecipitation buffer consisting of 50 mM Tris (pH 7.4), 100 mM NaCl, 3 mM MgCl_2_, IGEPAL 0.5%. RNA was extracted from Dynabeads using 1 mL TRIzol and 200 μL chloroform per sample. After the second round of TRIzol chloroform RNA purification, RNA precipitation was done using ice-cold isopropanol, sodium acetate, and GlycoBlue (Thermo Fisher) n ice for 1 hour. The RNA was resuspended in 7 μL of water. The RNA yields were approximately 6 ng/μL to 16 ng/μL for J2 antibody immunoprecipitation (lower in the nuclear fraction), while the isotype control yielded less than 300 pg/μL.

### A-to-I editing analysis.

The entire data processing scripts are available through GitHub: https://github.com/hato-lab/A-to-I-edit FASTQ files were initially aligned to the reference genomes: GRCm38 primary assembly and Gencode vM25 GTF files for mouse and GRCh38 and v41 GTF files for human, using the STAR aligner (v2.7.9a). To capture hyper-edited reads (59), we generated pseudo-genome references where all “A” bases were substituted with “G” (Supplemental Figure 11B). The unaligned reads from the initial alignment were realigned to the pseudo-genome reference using the STAR aligner with the same parameters (hyper-edited reads).

A-to-I editing sites were first detected using reditools2 extract_coverage.sh and parallel_reditools.py (82). The resulting A-to-I editing sites underwent additional filtering based on the following criteria: for a given editing site, there must be at least 3 samples with edited reads greater than 5 and an editing rate greater than 0.1 but less than 0.9 in order to reduce the inclusion of low editing loci and potential genomic mutations, respectively. The reading depth of the remaining sites (total counts) was obtained using the Samtools (83) depth command with the -b option (v1.9). Each A-to-I editing site was annotated using biomaRt (84) and a repeat class file obtained from the UCSC Genome Browser. Fisher’s exact test was used to compare edit ratio group comparisons. *P* values for each comparison were adjusted using the false discovery rate method. Sites with false discovery rate–adjusted *P* values less than 0.05 were considered significant for each comparison. ANNOVAR (85) was used to identify coding sequence mutations, and motif enrichment analysis was done using kpLogo (86). Complementary repeat region analysis was done using Biostrings:findPalindromes in R. RNAfold was used for the prediction of RNA minimum free energy secondary structures (87).

### Metabolomics.

Untargeted global metabolomic analysis was performed at Metabolon Inc. Snap-frozen mouse kidney tissues were processed following the Metabolon standard extraction method (60% methanol) and Metabolon’s ultra-performance liquid chromatography–tandem mass spectrometry pipeline. HPLC measurements were conducted in our laboratory using an Agilent 1100 series system equipped with a UV detector set at 254 nm, a fluorescence detector set at 335/510 nm, and a MilliporeSigma SUPELCOSIL LC-18-T column (15 cm × 4.6 mm with a particle size of 3 μm). For measurements of polyamines and polyamine precursors, tissues were homogenized using prechilled 10% HClO_4_. After centrifugation, 200 μL of each supernatant was slowly neutralized with 400 μL of NaHCO_3_ (8–9 g/100 mL at room temperature) (88, 89). Dansylation of polyamines/amino acids was done by addition of 800 μL of dansyl chloride in acetone (5 mg/mL), incubated at 70°C for 5 minutes. The reaction was terminated by addition of 200 μL of l-proline (100 mg/mL water) and incubated in the dark for 30 minutes at room temperature. Dansylated amino acids were extracted by addition of 100 μL of toluene, vortexed for 1 minute. After centrifugation, the organic phase (supernatant) was transferred to a new vial and concentrated for 10 minutes at 60°C (SpeedVac Concentrator SPD1010). The extract was dissolved in 300 μL of acetonitrile and filtered (0.45 μm PTFE MicroSpin filter, Chrom Tech). Buffer A was composed of acetonitrile/water (50/50 vol/vol), and buffer B was 100% acetonitrile. Our gradient elution program consisted of 0% B at 0 minutes, 0% to 15% linear gradient from 1 minute to 16 minutes, 50% to 80% from 16 minutes to 26 minutes, 26 to 28 minutes isocratic, 80% to 100% linear gradient from 28 minutes to 34 minutes.

### Statistics.

Data were analyzed for statistical significance and visualized with R software 4.1.0. Error bars show SD. For multiple comparisons, 1-way ANOVA followed by pairwise *t* tests was performed using the Benjamini and Hochberg method to adjust *P* values. All analyses were 2-sided, and *P* values less than 0.05 were considered significant.

### Study approval.

All animal protocols were approved by the Indiana University Institutional Animal Care and Use Committee and conformed to the NIH *Guide for the Care and Use of Laboratory Animals* (National Academies Press, 2011). Work with human subjects was approved by the Institutional Review Board of Indiana University School of Medicine (IRB 190657223). Tissues from the Biopsy Biobank Cohort of Indiana were acquired under waiver of informed consent. The Kidney Precision Medicine Project participants gave written informed consent.

### Data availability.

RNA-Seq data were deposited in the NCBI’s GEO database:

Human kidney biopsy RNA sequencing data are available at https://connect.posit.iu.edu/bulk_kidney_bx/

For dsRNA IP sequencing (LPS time course), see GSE244941. Representative read coverage tracks for the cytoplasmic fraction are available on a genome browser at https://connect.posit.iu.edu/view_GY/

For bulk kidney total RNA-Seq data for wild-type mice after LPS challenge (LPS time course), see GSE247727.

For bulk kidney total RNA-Seq data for wild-type mice after ischemia/reperfusion injury (IRI) (IRI time course), see GSE267650 (reviewer token: mdynmimctbahrcn). Data are available at https://connect.posit.iu.edu/IRI_timecourse_WT/

For bulk kidney total RNA-Seq data for Azin1-locked and uneditable mice (IRI time course), see GSE253286 for baseline and 24 hours and GSE267650 for 48 and 72 hours (reviewer token: mdynmimctbahrcn). Azin1 mouse kidney RNA-Seq data for baseline and 24 hours after IRI are available at https://connect.posit.iu.edu/azin1_mouse_kidney/

For mouse kidney nanopore PCR-free direct cDNA sequencing (LPS time course), see GSE244942 (reviewer token: otinkqkqdjypbav).

AZIN1 cell line RNA-Seq data are available at https://connect.posit.iu.edu/azin1/

Proteomics data are deposited in ProteomeXchange (accession MSV000093887; ID: MSV000093887_reviewer; password: Azin).

Reanalysis of Ribo-Seq and single-cell RNA-Seq was performed using GSE120877 and GSE151658. A Supporting Data Values file is provided as supplemental material.

### Code availability.

Scripts are available through GitHub: https://github.com/hato-lab/A-to-I-edit

## Author contributions

TH designed and coordinated the study. SH, CZ, AZ, SM, AN, QM, SCJ, EHD, BM, MH, and TH performed experiments. SH, JM, and TH performed data analyses. SH, PCD, and TH interpreted data. MTE and TMT provided clinical data and human kidney biopsy samples. PCD and TH wrote the manuscript.

## Supplementary Material

Supplemental data

Unedited blot and gel images

Supporting data values

## Version 1 07/02/2024

In-Press Preview

## Version 2 09/03/2024

Electronic publication
