# Supplementary material for: Inflammation primes the murine kidney for recovery by activating AZIN1 adenosine-to-inosine editing
Source: J Clin Invest. 2024 Sep 3;134(17):e180117. doi: 10.1172/JCI180117 (PMC11364396; doi:10.1172/JCI180117)
Supplement: Supplemental data [file jci-134-180117-s118.pdf]

Supplemental Material

Inflammation primes the murine kidney for recovery by activating AZIN1 adenosine-to-inosine editing

Segewkal Heruye[1], Jered Myslinski[1], Chao Zeng[2], Amy Zollman[1], Shinichi Makino[1], Azuma Nanamatsu[1], Quoseena Mir[3], Sarath Chandra Janga[3], Emma H Doud[4], Michael T Eadon[1], Bernhard Maier[1], Michiaki Hamada[2,5,6], Tuan M Tran[1,7], Pierre C Dagher[1], Takashi Hato[1,7,8*]

[1]Department of Medicine, Indiana University School of Medicine

[2]Faculty of Science and Engineering, Waseda University, Tokyo

[3]Luddy School of Informatics, Computing, and Engineering, Indiana University

[4]Department of Biochemistry and Molecular Biology, Indiana University School of Medicine

[5]AIST-Waseda University Computational Bio Big-Data Open Innovation Laboratory, National Institute of Advanced Industrial Science and Technology, Tokyo

[6]Graduate School of Medicine, Nippon Medical School, Tokyo

[7]Richard L. Roudebush Veterans Affairs Medical Center, Indianapolis

[8]Department of Medical and Molecular Genetics, Indiana University School of Medicine

*Correspondence: thato@iu.edu

Supplemental Methods

dsRNA UV crosslinking and immunoblotting

Total RNA extraction was done on mouse kidney lysate using the RNeasy Plus Universal Midi kit (Qiagen). RNase digestion was then performed on the RNA extract using the following RNases. RNase A (Thermo Fisher EN0531) cleaves single-stranded RNA when NaCl is greater than 0.3 M. RNase A (1.5 µg in 1.5 µl) was added to 3.5 µL of 5M NaCl and 15 µg of RNA extract in a total volume of 50 µl and incubated at room temperature for 10 min. RNase III (Thermo Fisher AM2290) cleaves double-stranded RNA. RNase III (15 U in 15 µl) was mixed with 15 µg of RNA extract in 5 µl of 10X RNase III reaction buffer consisting of 500 mM NaCl, 100 mM Tris pH 7.9, 100 mM $MgCl_2$, 10 mM DTT, brought up to a total volume of 50 µl, and incubated at 37 °C for 1 hour. For both RNase A and RNase III digestion, the reaction was deactivated by adding 300 µl of TRIzol. Digested RNA was purified using Direct-zol (Zymo Research), and then 20 units of Superase-In was added to each sample (Thermo Fisher) before preparing a serial dilution. As a positive control, double-stranded RNA poly(I:C) (Invivogen) was used. An equal volume (2.5 µl) of purified RNA was dotted on Amersham Hybond N+ membrane (VWR) and crosslinked using an UV Stratalinker 2400 (Autocrosslink mode). Odyssey Blocking buffer was used for blocking. The membrane was incubated with anti-dsRNA monoclonal antibody J2 (SCICONS/Jena Bioscience RNT-SCI-10010200) at a concentration of 2.5 µg/mL overnight at 4 °C followed by anti-mouse secondary antibody.

Illumina short-read RNA sequencing

Stranded total RNA-sequencing was performed at the Indiana University Center for Medical Genomics Core. cDNA library preparation was carried out using the Clontech SMARTer Stranded Total RNA-Seq Kit v2. The pooled libraries were loaded onto a NovaSeq 6000 sequencer at 300 pM final concentration for 100 bp paired-end sequencing (Illumina). Approximately 40 million reads per library was generated. Phred quality score (Q score) was used to measure the quality of sequencing. More than 90% of the sequencing reads reached Q30 (99.9% base call accuracy). The sequenced data were mapped to the mm10 or hg38 genome using STAR. The counts data were generated using featureCounts and analyzed using edgeR (https://github.com/hato-lab/A-to-I-edit). P values were adjusted with the FDR method as indicated. Gene set enrichment analysis was done using the R package fsgea (Gene set co-regulation analysis) and Molecular Signatures Database Hallmark Gene Sets.

Nanopore long-read RNA sequencing

Snap-frozen mouse kidney tissue was homogenized in 800 µl of Tri Reagent using a Minilys tissue homogenizer at the highest speed for 45 seconds and then incubated for 5 minutes. Total RNA was extracted from 600 µl of the supernatant using the Direct-zol RNA miniprep Plus (Zymo Research), including on-column DNase I digestion. The RNA was eluted in 100 µl of water and subjected to mRNA polyA enrichment using the Dynabeads mRNA DIRECT Micro kit

(Thermo Fisher 61021). After two rounds of washing and enrichment of mRNA following the manufacture's protocol, polyA+ mRNA was eluted in 10 μl of water.

To establish our Nanopore RNA sequencing workflow, we initially compared direct RNA sequencing (SQK-RNA002), direct cDNA sequencing (SQK-DCS109), and cDNA-PCR sequencing (SQK-PCS111), all on R9.4.1 flow cells. We chose direct cDNA sequencing because: 1) direct RNA sequencing had the lowest read coverage and 3' reads bias, and 2) cDNA-PCR sequencing had the shortest reads length (but the highest reading depth). We proceeded with approximately 200 ng of polyA+ mRNA as the starting material. Reverse transcription and strand-switching was done using Maxima H Minus Reverse Transcriptase following the Nanopore protocol (SQK-DCS109). After the second strand synthesis, the RNA concentration was generally 20-30 ng/μl as measured by NanoDrop. Following end-prep, adapter ligation, and AMPure XP bead binding, the yield was generally 6-10 ng/μl as measured by Qubit. Sequencing was conducted on R9.4.1 flow cells using the GridIon platform.

The basecalling was done using Guppy basecaller:

```
guppy_basecaller --compress_fastq --fast5_out -i ./fast5_pass/ -s ./fastq/ --device 'auto' --num_callers 1 --flowcell "FLO-MIN106" --kit "SQK-DCS109"
```

Mapping was carried out using Minimap2

```
minimap2 -a -L -t 12 -x splice --junc-bed Mus_musculus.GRCm38.101.bed --MD mm10-ont.mmi ./fastq/*.fastq.gz 2> ./minimap2.err > ./SAM/$sample.sam
```

Subsequent transcript-level analysis was performed using Bambu (1).

Transformation of E. coli and plasmid transfection of HEK293T cells

We designed the following two plasmids based on the pCDH-EF1a-eFFly-mCherry backbone vector (Addgene #104833) with cloning sites BmtI and BamHI. Gene synthesis was done by GenScript, and the resulting plasmid sequences were verified using Sanger sequencing.

pCDH-EF1a-3xFLAG-AZIN1_locked-T2A-mCherry

pCDH-EF1a-3xFLAG-AZIN1_uneditable-T2A-mCherry

The sequence of 3xFLAG tag consisted of: gccaccatg(Kozak sequence)gactacaaagaccatgacggtgattataaagatcatgacatcgattacaaggatgacgatgacaagAAA(AZIN1 CDS in frame).

The sequences of AZIN1-locked and uneditable plasmids differ at the A-to-I editing site, following the same mutation strategy used in the CRISPR knock-in cell lines.

A-to-I locked: 5'TGATGAGCTTGATCAAATTGT**c**GAA**g**GC3'

A-to-I uneditable: 5'TGATGAGCTTGATCAAATTGT**c**GAA**tc**C3'

The stop codon of AZIN1 is removed and fused in frame with T2A and mCherry CDS as follows: GCT(end of AZIN CDS without a stop

codon)ggatccgcggccgctgagggcagaggaagtcttctaacatgcggtgacgtggaggagaatcccggccctatgcatATG(
mCherry CDS start).

Chemically competent E. coli (One Shot Top10, Invitrogen C404003) were transformed with pCDH plasmids using the heat shock method (on ice for 30 min followed by 42 °C heat shock for 45 seconds). Transformed E. coli were incubated overnight in LB broth with 100 µg/mL carbenicillin at 225 rpm, 37 °C. Plasmid isolation was done using Nucleobond Xtra Midi Kit (Takara #740410.50) following the manufacture's protocol. For DNA gel electrophoresis, 2% agarose gel was prepared using TopVision Agarose Tablets (Thermo Fisher R2801) and ethidium bromide (Thermo Fisher 15585011). Samples were electrophoresed with NEB X6 gel loading dye in 0.5X TAE buffer with a TrackIt 100 bp DNA ladder at 100 V. Transfection of plasmids was done using lipofectamine 2000 following the manufacturer's instruction (sequential mixing of lipofectamine, Opti-MEM and plasmid into freshly replaced DMEM/10% FBS medium, Thermo Fisher 11668027). For all experiments, either 4 µg or 20 µg plasmid DNA was used per well of a 6 well-plate or 10 cm dish, respectively.

AZIN1 immunoprecipitation and mass spectrometry

Cells were cultured in 10-cm plates to 80% confluency and lysed using 1 ml of 1X dilution Cell Signaling Cell lysis buffer (#9803), which consists of 2 mM Tris-HCl (pH 7.5), 15 mM NaCl, 0.1 mM Na_2EDTA, 0.1 mM EGTA, 0.1% Triton, 0.25 mM sodium pyrophosphate, 0.1 mM beta-glycerophosphate, 0.1 mM Na_3VO_4, 0.1 µg/ml leupeptin, 1 mM PMSF (Thermo Fisher 36978), phosStop (1 pill per 10 ml), and protease inhibitor (1 pill per 10 ml; cOmplete Mini EDTA-free, Roche Diagnostics). The lysates were sonicated and then centrifuged at 14,000 x *g* for 10 minutes at 4°C. In a separate experiment, nuclear and cytoplasmic fractionation was done using 1 mL Pierce IP Lysis Buffer (87788). The cell suspension was passed through a 27-gauge needle 10 times using a 1mL syringe and incubated on ice for 20 minutes. The lysed cell suspension was then centrifuged at 720 x g at 4°C for 5 minutes. The resulting supernatant was collected as the cytoplasm fraction. The pellet was resuspended with 1 mL Pierce IP Lysis Buffer and passed through a 25-gauge needle 10 times, followed by centrifugation at 21,000 x g at 4°C for 15 min. The resulting supernatant was collected as the nuclear fraction.

FLAG-tag immunoprecipitation was performed by incubating 900 µL of the supernatant with 50 µL of pre-washed Pierce DYKDDDDK Magnetic Agarose (12.5 µL settled magnetic agarose; Thermo Fisher A36797) at room temperature for 20 minutes. The beads were washed three times with 1mL of phosphate buffered saline followed by urea denaturation, TCEP reduction, CAA alkylation, and trypsin/LysC digestion (Promega V5072). After digestion, samples were quenched with 2% formic acid. Peptides were separated on an Ultimate 3000 HPLC with loading on a 5 cm C18 trap column Acclaim PepMap 100 (3 µm particle size, 75 µm diameter; Thermo Scientific, 164946) followed by a 15 cm PepMap RSLC C18 EASY-Spray column (Thermo Scientific, ES900) and analyzed using a Q-Exactive Plus mass spectrometer (Thermo Fisher) operated in positive ion mode. Solvent B was increased from 5%-35% over 75 min, to 90% over 2 min, back to 3% over 2 minutes (Solvent A: 95% water, 5% acetonitrile, 0.1% formic acid; Solvent B: 100% acetonitrile, 0.1% formic acid). A data dependent top 15 method was used with MS scan range of 200-2000 m/z, resolution of 70,000, AGC target 3e6, maximum IT

of 100 ms. MS2 resolution of 17,500, fixed first mass 100 m/z, normalized collision energy of 30, isolation window of 4 m/z, target AGC of 1e5, and maximum IT of 50 ms. Dynamic exclusion of 30 sec, charge exclusion of 1, 7, 8, >8 and isotopic exclusion parameters were used. Data were analyzed by Proteome Discoverer 2.5 and SEQUEST HT. A *Homo sapiens* reviewed protein database was downloaded from the Universal Protein Knowledgebase / Translated European Molecular Biology Laboratory (051322 and supplemented with frequently observed contaminants for a full tryptic search with a maximum of 3 missed cleavages). Precursor mass tolerance was set to 10 ppm and fragment mass tolerance set at 0.02 Da. Dynamic peptide modifications were oxidation on methionine, phosphorylation on serine, threonine, or tyrosine; acetylation, met-loss, and met-loss plus acetylation at protein N-terminus. Static modifications were carbamidomethylation on cysteines. Percolator false discovery rate filtration of 1% was applied to both the peptide-spectrum match and protein levels. Search results were loaded into Scaffold 5 for visualization and analysis.

Polyribosomal profiling

For polyribosome profiling of tissues, cardiac perfusion was performed with 6 mL of cycloheximide (100 μg/ml in PBS, Sigma). Harvested tissues were immediately placed in a lysis buffer consisting of 1% Triton X-100, 0.1% deoxycholate, 20 mM Tris-HCl, 100 mM NaCl, 10 mM $MgCl_2$, EDTA-free Protease Inhibitor Cocktail Tablet (Roche) and 100 μg/ml cycloheximide. Tissues were homogenized using a Minilys tissue homogenizer (Bertin Instruments). Tissue homogenates were incubated on ice for 20 minutes, then centrifuged at 9,600 *g* for 10 minutes. The supernatant was added to the top of a sucrose gradient generated by BioComp Gradient Master (10% sucrose on top of 50% sucrose in 20 mM Tris-HCl, 100 mM NaCl, 5 mM $MgCl_2$, and 100 μg/ml cycloheximide) and centrifuged at 283,800 *g* for 2 hours at 4 °C. The gradients were harvested from the top in a Biocomp harvester (Biocomp Instruments), and the RNA content of eluted ribosomal fractions was continuously monitored with UV absorbance at 254 nm. For polyribosome profiling of cultured cells, cells were rinsed with cold PBS first, then cells were scraped using the lysis buffer on ice. The remaining procedure was identical to tissue polyribosome profiling.

Seahorse bioenergetics assays

First, Seahorse XFp cell energy phenotype test (Agilent) was performed. The stressor mix recipe consisted of FCCP and oligomycin, each at a well concentration of 1 μM. After confirming that HEK293T cells, including AZIN1 A-to-I locked and uneditable mutants, are glycolytic, we proceeded to the XFp glycolysis stress test. Cells were seeded 24 hours prior to the stress test (8,000 cells in 100 μl of DMEM per well). A glucose-free assay medium was prepared by mixing 10 ml of Seahorse XF base medium minimal DMEM (Agilent 103334-100), 100 μl Na pyruvate (Sigma S8636), and 200 μl L-glutamine (gibco 25030-081), and the pH was adjusted to 7.4 using NaOH. The medium was replaced with the glucose-free assay medium following the Seahorse protocol. Port A consisted of glucose (final well concentration 10 mM), Port B contained oligomycin (2 μM), and Port C contained 2-deoxyglucose (50 mM).

Real-time cell growth monitoring

Cells were seeded on the first day of live cell imaging at a density of 2,500 cells per well or 5,000 cells per well in 100 µl of DMEM on a 96-well plate. The Sartorius IncuCyte system was used with either a 4X or 10X objective lens, and 2- or 4-hour imaging intervals for 5 days. In some experiments, urea (Sigma U5378) was added at a well concentration of 60 mM. DMEM SILAC medium (Thermo Fisher 88364) and DMEM without glutamine (Thermo Fisher 10313021) were used for indicated experiments. Cell growth quantitation was performed using the IncuCyte built-in masking algorithm.

PCR

RNA was extracted using TRI Reagent and Direct-zol RNA MiniPrep Plus (Zymo Research R2070). Reverse transcription was done using High Capacity cDNA Reverse Transcription kit (ThermoFisher/Applied Biosystems 4368814). Conventional PCR was done using 1.8% agarose gel. PCR primers used include:

MuERV 5'TTTCTCAAGGCCCACCAATAGT3' (forward) and 5'GACACCTTTTTAACTATGCGAGCT3' (reverse) (2)

MusD 5'GATTGGTGGAAGTTTAGCTAGCAT3' (forward) and 5'TAGCATTCTCATAAGCCAATTGCAT3' (reverse) (3)

Line1 5'TTTGGGACACAATGAAAGCA3' (forward) and 5'CTGCCGTCTACTCCTCTTGG3' (reverse) (3)

IAP 5'CTTGCCCTTAAAGGTCTAAAAGCA3' (forward) and 5'GCGGTATAAGGTACAATTAAAAGATATGG3' (reverse) (3)

Western blotting

Proteins from cells and tissues were extracted using RIPA buffer (ThermoFisher Pierce) with 0.5M EGTA, 0.5M EDTA, DNase I (0.1 U/µL, Ambion AM2222), Halt protease inhibitors (Pierce), phosStop inhibitor (Roche), and benzonase nuclease (25 U/mL EMDMillipore 70746-4). Total protein levels were determined using a modified Lowry assay (Bio-Rad). Equal amounts of total proteins (10 - 20 µg) were mixed with NuPAGE LDS Sample Buffer (Thermo Fisher) containing 100 mM DTT and separated by electrophoreses on NuPage 4%–12% Bis-Tris gels, followed by transfer to PVDF membranes. Samples related to immunoprecipitation (input, flowthrough, and immunoprecipitated proteins) were lysed with Pierce IP Lysis Buffer (25 mM Tris-HCl pH 7.4, 150 mM NaCl, 1 mM EDTA, 1% NP-40, 5% glycerol). For AZIN1 protein turnover determination, cells were incubated with 20 µM MG132 for a total of 3 hours and 250 µg/ml cycloheximide for the indicated duration before cell lysis. Antibodies used include the following: Antizyme inhibitor 1 (Proteintech, rabbit polyclonal, 1:1,000 dilution, #11548-1-AP), Insulin degrading enzyme (9B12.225, BioLegend, mouse monoclonal, 1:500 dilution, #921203), β-actin (AC-15, Invitrogen, mouse monoclonal, 1:3,000 dilution, #MA1-91399), Histone H3 (1B1B2, Cell Signaling, mouse monoclonal, 1:1,000 dilution, #14269), Alexa Fluor 680

AffiniPure donkey anti-rabbit and Alexa Fluor 790 AffiniPure donkey anti-mouse secondary antibodies (Jackson ImmunoResearch, 1:5,000 dilution #715-655-150, #711-625-152). Quantitation was done using Image Studio.

Immunohistochemistry

Tissues were fixed with 4% paraformaldehyde, sectioned at 5 μm, and deparaffinized. Low pH antigen retrieval was used. Tissues were stained for Spermidine using anti-spermidine antibody (Abcam ab7318, 1:100 dilution) and imaged using a Leica DM 1000 LED. Hematoxylin and eosin-stained kidney sections were graded for ischemia injury (casts, tubular dilation, tubular necrosis, and interstitial edema) as previously described (4). Congo red stain for amyloid β was performed on deparaffinized brain sections.

Flow cytometry

Femur bone marrow cells were collected in 1% FBS/PBS. After Fc blocking with anti-mouse CD16/32 (clone 93), cells were incubated with the following antibodies: APC anti-mouse CD45 (clone 30-F11), BV605 anti-mouse/human CD11b (clone M1/70), FITC anti-mouse CD4 (clone RM4-5), BV785 anti-mouse CD8a (clone 53-6.7), and PerCP-Cy5.5 anti-mouse/human B220/CD45R (clone RA3-6B2), all from BioLegend. Red Blood Cell Lysing Buffer Hybri-Max (Sigma R7757) and propidium iodide were used to exclude red blood cells and dead cells, respectively. For intracellular cytokine staining, cells were first stained with CD45, CD11b, and B220 antibodies. After fixation and permeabilization (Cyto-Fast Fix/Perm Buffer Set, BioLegend cat. 462803), cells were incubated with the following antibodies: BV785 anti-mouse TNF-α (clone MP6-XT22), FITC anti-mouse IL-10 (clone JES5-16E3), and PE anti-mouse IL-6 (clone MP5-20F3). Analysis was done using a CytoFLEX Beckman Coulter Flow cytometer.

Statistics

Data were analyzed for statistical significance and visualization with *R* software 4.1.0. Error bars show SD. For multiple comparisons, 1-way ANOVA followed by pairwise *t* tests was performed using the Benjamini and Hochberg method to adjust *P* values. All analyses were 2-sided, and a *P* value of less than 0.05 was considered significant.

References

1. Chen Y, Sim A, Wan YK, Yeo K, Lee JJX, Ling MH, et al. Context-aware transcript quantification from long-read RNA-seq data with Bambu. *Nat Methods.* 2023;20(8):1187-95.
2. Kigami D, Minami N, Takayama H, and Imai H. MuERV-L is one of the earliest transcribed genes in mouse one-cell embryos. *Biol Reprod.* 2003;68(2):651-4.
3. Rowe HM, Jakobsson J, Mesnard D, Rougemont J, Reynard S, Aktas T, et al. KAP1 controls endogenous retroviruses in embryonic stem cells. *Nature.* 2010;463(7278):237-40.
4. Dominguez JM, 2nd, Dominguez JH, Xie D, and Kelly KJ. Human extracellular microvesicles from renal tubules reverse kidney ischemia-reperfusion injury in rats. *PLoS One.* 2018;13(8):e0202550.

Supplemental Figure 1

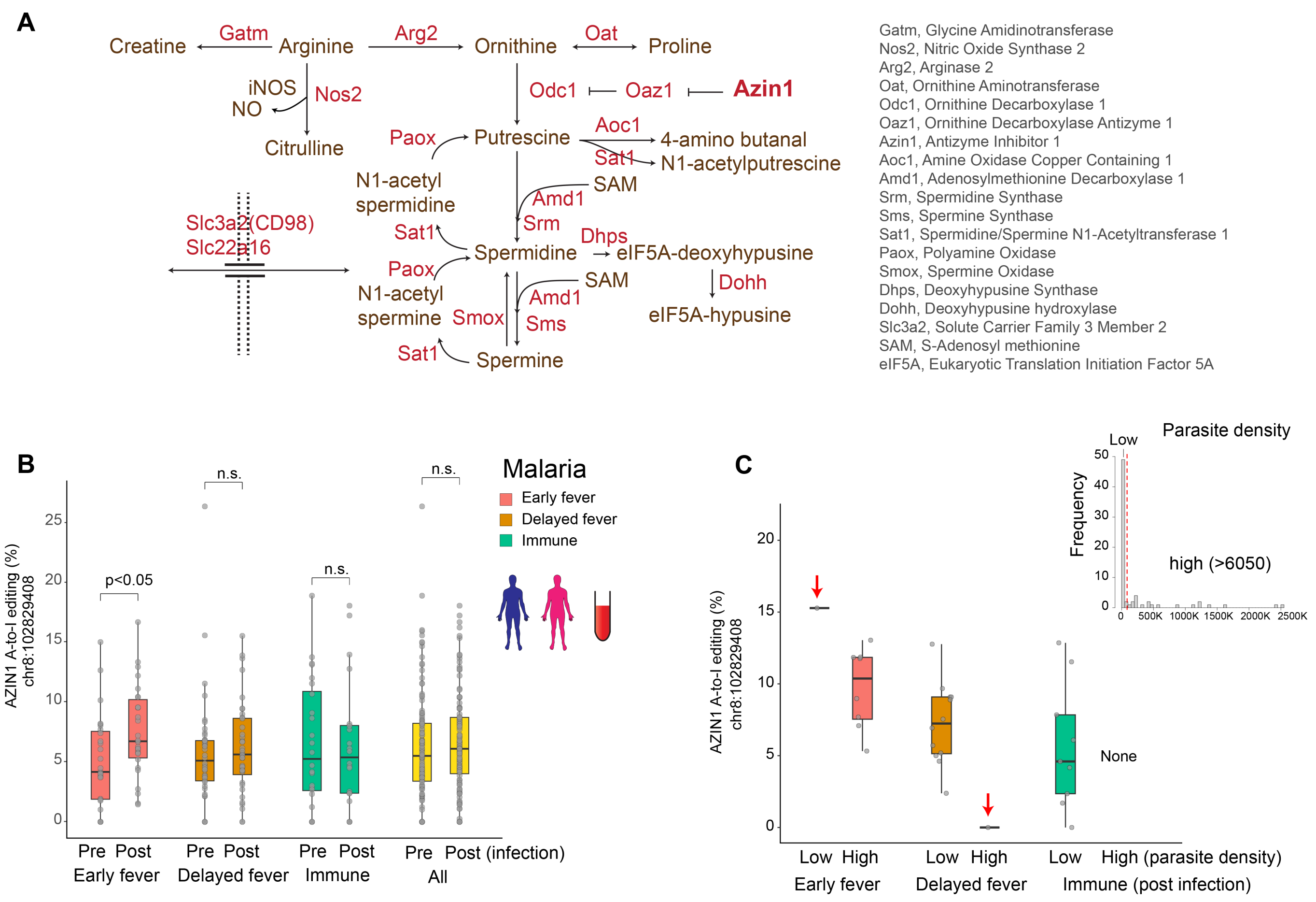

Supplemental Figure 1

(**A**) Depiction of key molecules involved in the polyamine pathway. (**B**) Distribution of AZIN1 A-to-I editing rates (% of edited reads over total reads) for combined male and female individuals. (**C**) Distribution of AZIN1 A-to-I editing rates based on parasite density and clinical category for male children after malaria infection. The early fever group exhibited high parasite density, except for one child who had the highest AZIN1 editing rate and low parasite density. The delayed fever group had low parasite density, except for one child who had the lowest AZIN1 editing rate and high parasite density. All immune children had low parasite density.

Supplemental Figure 2

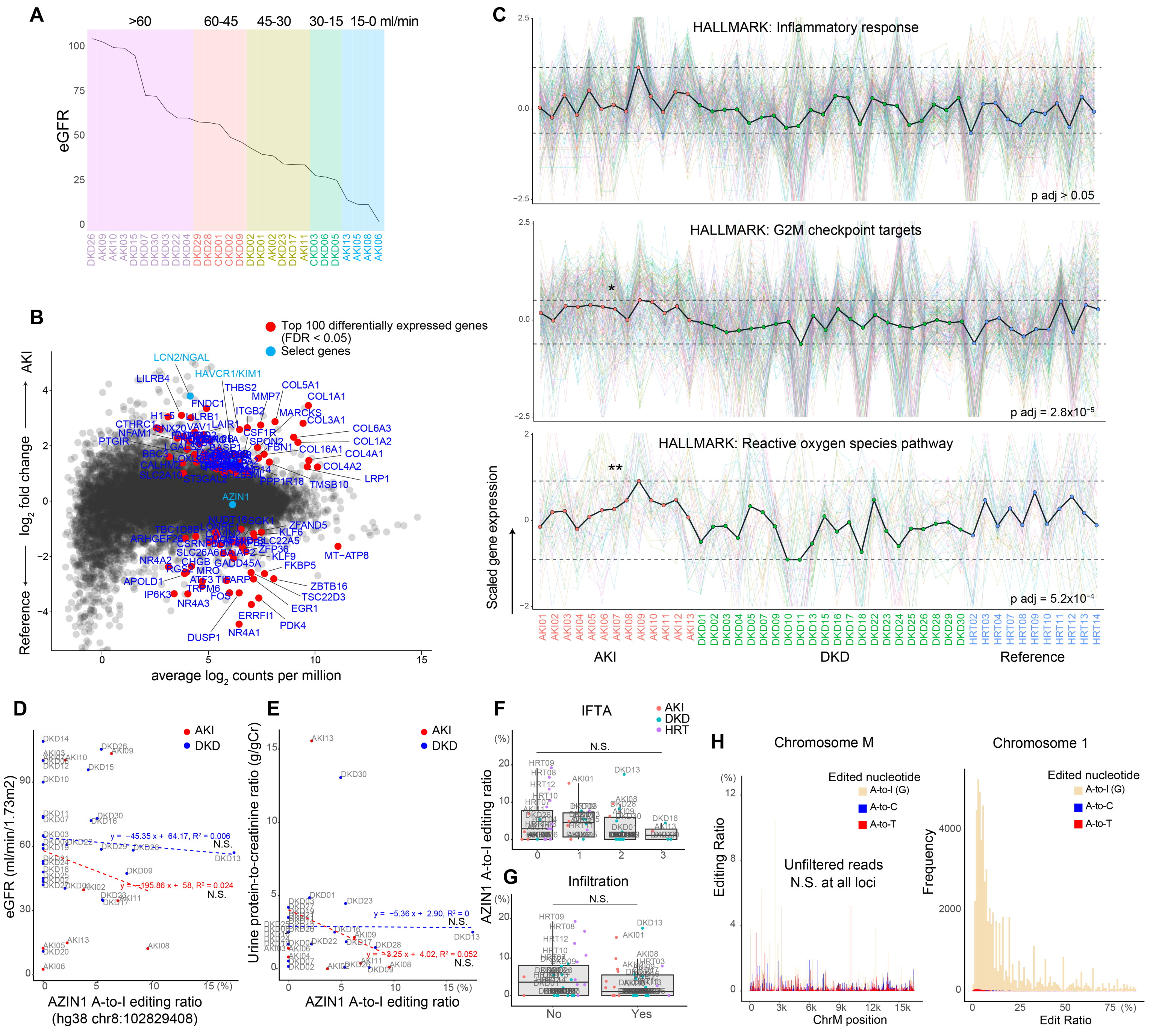

Supplemental Figure 2
(**A**) Line plot of indicated conditions sorted by estimated glomerular filtration rate (eGFR). Detailed clinical data are not available, and the timing of kidney biopsy and the eGFR measurement is unclear. (**B**) RNA-seq gene expression analysis (https://connect.posit.iu.edu/bulk_kidney_bx/). Smear plot highlighting the top 100 differentially expressed genes in red (AKI versus reference human biopsies). (**C**) Gene set co-regulation analysis showing inter-individual heterogeneity (select pathways are shown using Molecular Signatures Database Hallmark gene sets). One-way analysis p-values are indicated in each panel. *Pairwise t-test adjusted $p < 0.05$ compared with DKD or Reference, **$p < 0.05$ compared with DKD. (**D**-**G**) Plots showing the relationships between the rate of AZIN1 A-to-I editing and indicated clinical or pathology attributes. IFTA, interstitial fibrosis and tubular atrophy. Infiltration denotes immune cell infiltration as recorded in pathology reports. (**H**) Bar plot showing the lack of mitochondrial A-to-I editing (left). For clarity, all unfiltered reads, including regions with low read coverage, are shown. None of the site reached statistical significance. Note the mixture of A-to-N, rather than enrichment of A-to-I, and low levels of editing (i.e., noise). As a reference, representative A-to-I editing observed in a non-mitochondrial chromosome is shown on the right. The histogram depicts the enrichment of A-to-I editing.

Supplemental Figure 3

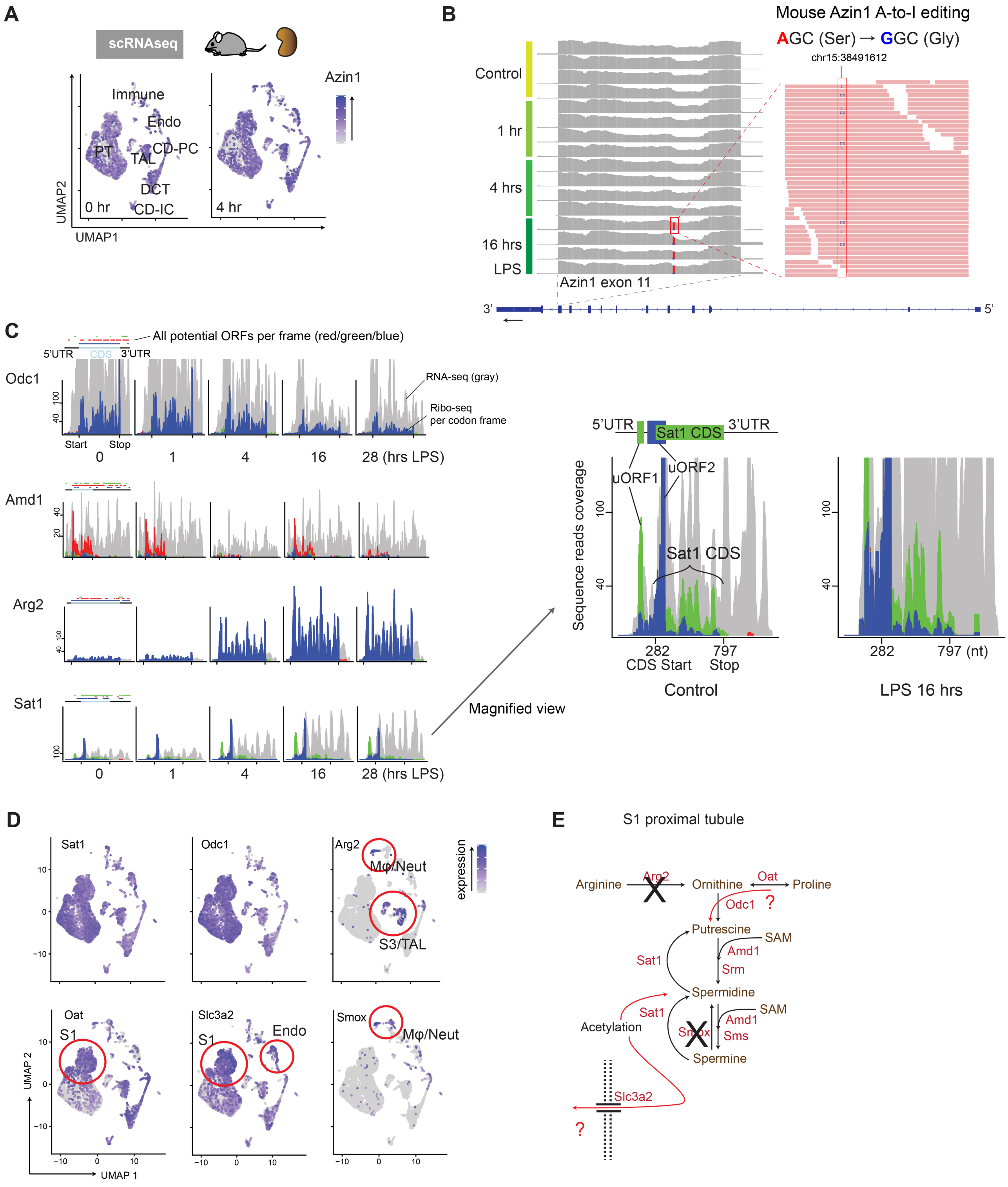

Supplemental Figure 3

(**A**) Single-cell UMAP displaying the diffuse distribution of Azin1 expression in all cell types in the kidney (Reanalysis of published data GEO GSE151658). (**B**) Total RNA-seq read coverage around the Azin1 A-to-I editing site in the mouse kidney tissues under the indicated conditions. A magnified view at 16 hours after LPS is shown for one sample. (**C**) Combined Ribo-seq and RNA-seq read coverage graphs for the indicated genes (ENSEMBL Odc1-201, Amd1-201, Arg2-201, Sat1-201). Odc1 and Amd1 genes are polyamine anabolic enzymes, whereas Sat1 is a polyamine catabolic enzyme. (**D**) Single-cell UMAP displaying gene expression levels and the distribution of indicated transcripts in the kidney. Select cell types are highlighted with red circles. (Smox and Oat are from 4 hours after LPS, and Arg2 is from 36 hours after LPS, the rest at from 0-hour baseline). (**E**) Interpretation of the single-cell RNA-seq data. S1 proximal tubules may preferentially utilize proline as a precursor for polyamines, whereas S3 proximal tubules rely more on arginine.

Supplemental Figure 4

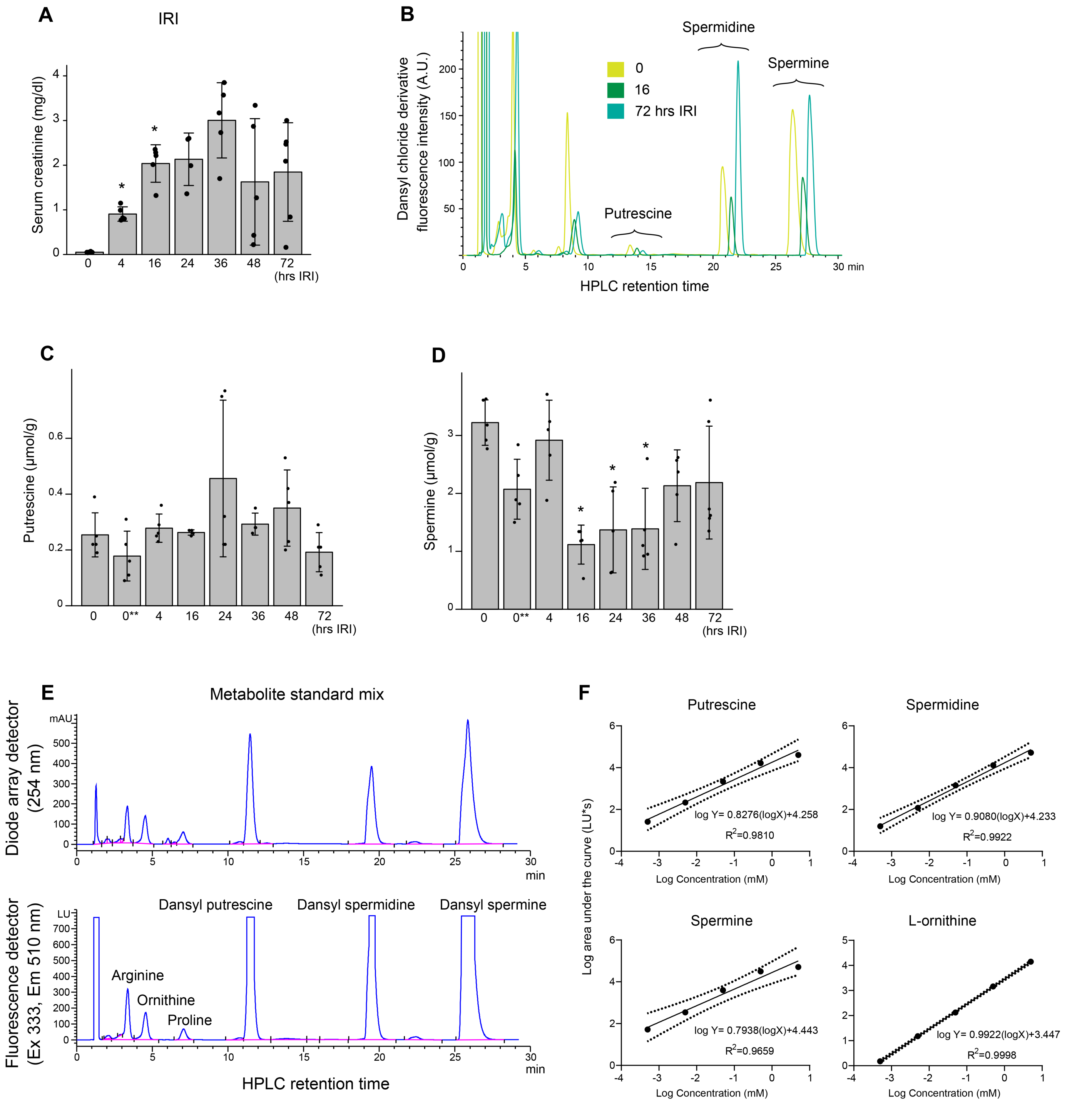

Supplemental Figure 4

(**A**) Serum creatinine levels at indicated time points after 20-minute bilateral ischemia-reperfusion injury in C57BL/6J male mice. (**B** - **D**) Measurements of kidney tissue polyamine levels by HPLC under indicated conditions. Representative chromatograms are also shown. For clarity, the traces are slightly shifted from each other on the x-axis elution time. *$p < 0.05$ vs 0-hour control samples, 1-way ANOVA followed by Dunnett's test for multiple treatment comparisons. 0** indicates kidney tissues harvested 20 minutes after ischemia without reperfusion. (**E** – **F**) Chromatograms of dansyl-derivatized polyamine standards mix and dose response curves of standards are shown as quality metrics for the HPLC polyamine quantitation assay.

Supplemental Figure 5

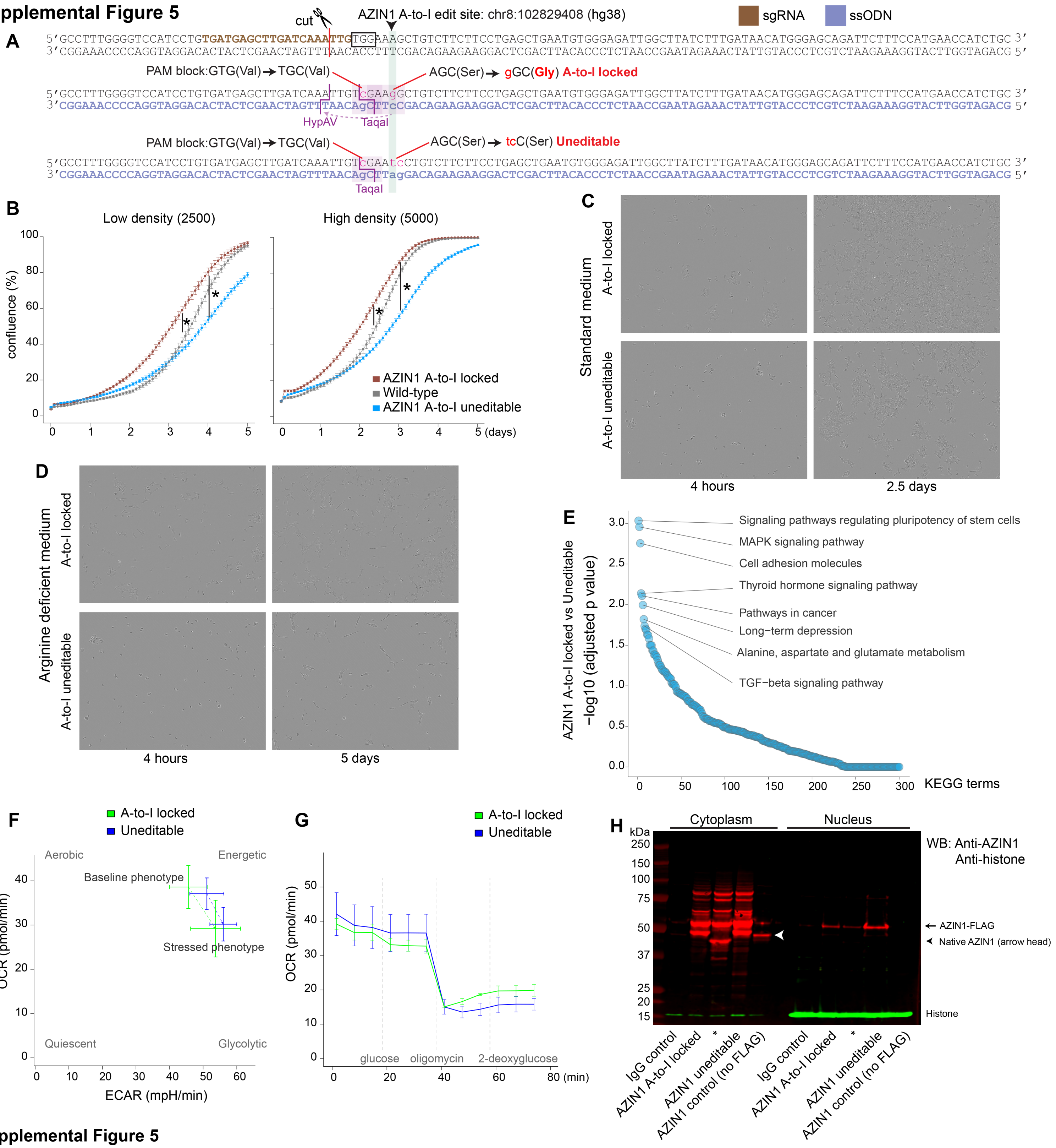

Supplemental Figure 5

(**A**) CRISPR knock-in design used for generating the Azin1 A-to-I locked and uneditable cell lines. The single guide RNA is shown in brown, and single-stranded oligonucleotides are shown in blue. Key mutations introduced are annotated. (**B**) Comparison of cell growth between low-density and high-density seeding for AZIN1 A-to-I locked, uneditable, and wild-type cells (IncuCyte, 10x objective lens, imaged every 2 hours for 5 days. For clarity, high-density seeding data shown in the Main Figure 3D are presented again). *p<0.05 at all time points for the indicated conditions, except for day 1 and the stationary phase between AZIN1 A-to-I locked and wild-type cells. (**C**, **D**) Representative time-lapse images under the indicated conditions. A-to-I uneditable cells exhibit delayed adherent cell morphology (compared at 4 hours post cell seeding). (**E**) KEGG pathway enrichment analysis comparing AZIN1 A-to-I locked and uneditable cell lines (RNA-seq data, n=3 technical replicates for each cell type). Approximately 300 KEGG metabolic pathway terms are aligned in the order of statistical significance.
(**F**) Seahorse cell energy phenotype test. The x-axis denotes extracellular acidification rates, and y-axis denotes oxygen consumption rates. (**G**) Oxygen consumption rates corresponding to the Main Figure 3H Seahorse glycolysis stress test, confirming the effect of oligomycin in both cell lines. n=3 independent experiments with n=3 technical replicates for each experiment. (**H**) Western blotting for AZIN1 and histone, showing the expected shift of AZIN1 due to FLAGx3 as well as differential expression of histone between cytoplasmic and nuclear fractions. *denotes a plasmid construct not used in this manuscript.

Supplemental Figure 6

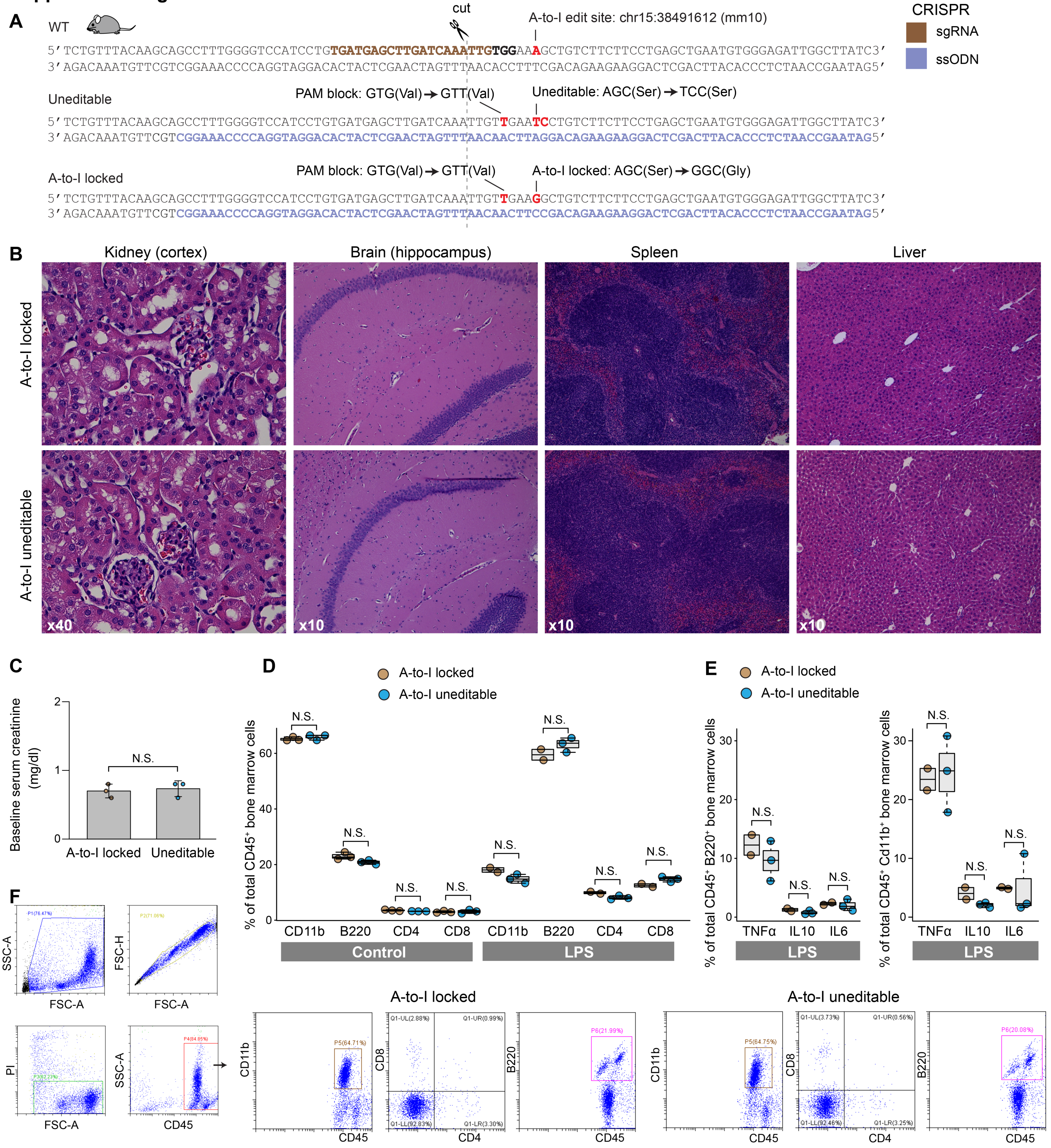

Supplemental Figure 6

(**A**) CRISPR knock-in design used for generating the Azin1 A-to-I locked and uneditable mice. The single guide RNA is shown in brown, and single-stranded oligonucleotides are shown in blue. Key mutations introduced are annotated. (**B**) Hematoxylin and eosin staining of the indicated organs under normal conditions in Azin1 A-to-I locked and uneditable mice. (**C**) Baseline serum creatinine levels of Azin1 A-to-I locked and uneditable mice are shown. (**D**) Flowcytometric analysis of bone marrow cells under normal conditions and 16 hours after LPS intravenous injection in Azin1 A-to-I locked and uneditable mice. (**E**) Percentage of indicated cytokine-expressing bone marrow cells 16 hours after LPS injection. (**F**) Representative flow cytometry dot plots are shown (baseline control samples).

Supplemental Figure 7

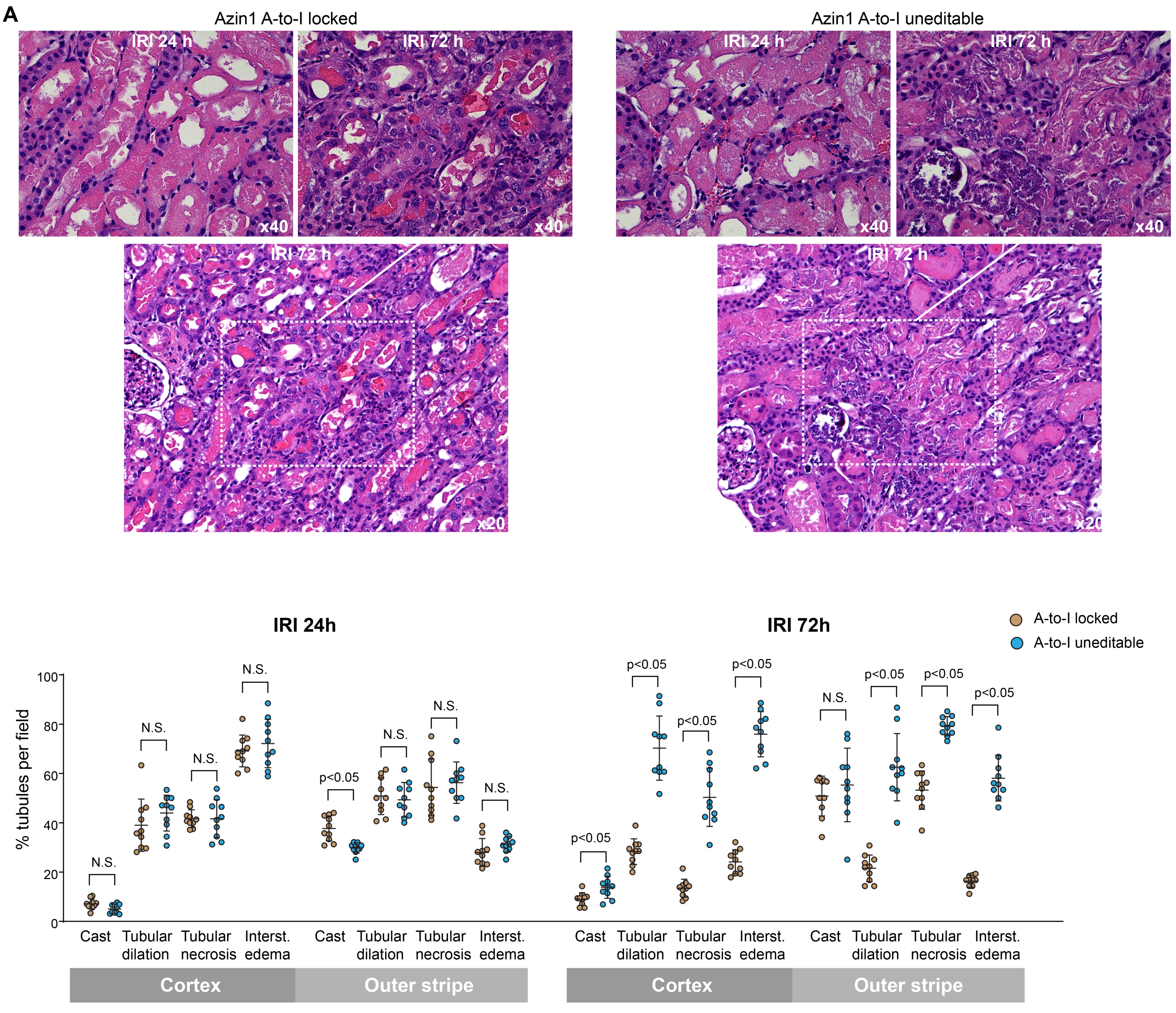

Supplemental Figure 7
(**A**) Hematoxylin and eosin staining 24 and 72 hours after renal ischemia-reperfusion injury. The degree of injury is quantitated under the indicated conditions (1-way ANOVA followed by a t-test with Bonferroni correction for multiple treatment comparisons).

Supplemental Figure 8

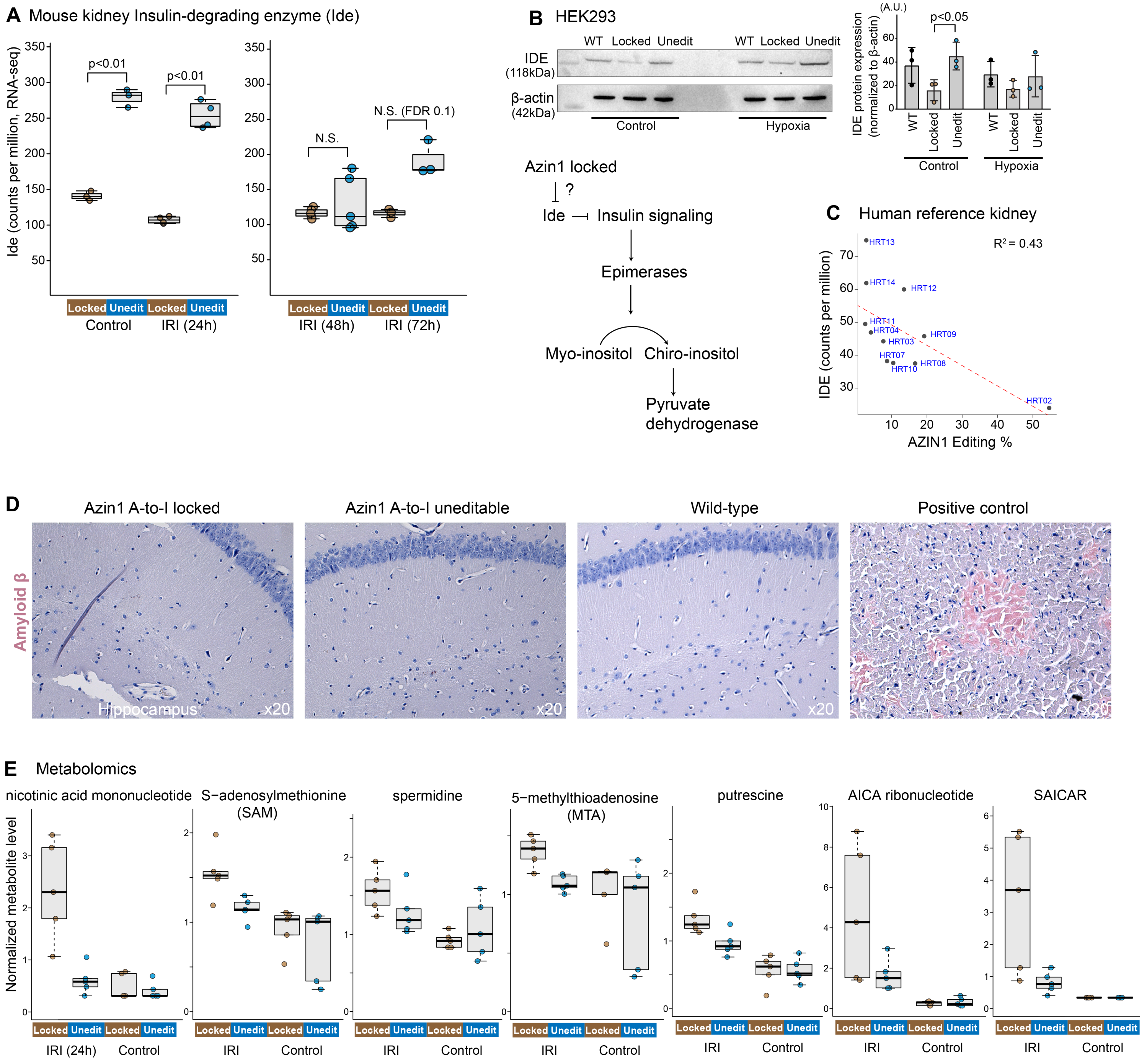

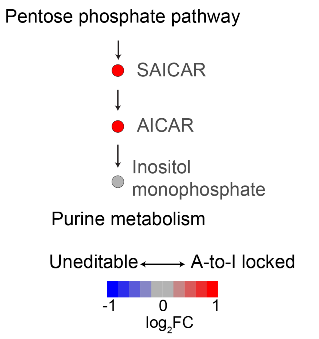

Supplemental Figure 8
(**A**) RNA-seq read counts are shown for Ide (Insulin-degrading enzyme) under indicated conditions. The left panel shows Ide transcript counts for control and IRI at 24 hours. The right panel shows counts for IRI at 48 and 72 hours. These data were sequenced separately, hence the use of two separate panels. (**B**) Western blotting of HEK293 wild-type and AZIN1 mutant cells under normal culture conditions and after 3 hours of hypoxia (n=3 independent experiments). (**C**) Scatter plot of human reference kidneys depicting the editing percentage for AZIN1 and IDE gene expression levels. (**D**) Amyloid β staining under indicated conditions. (E) Select examples of metabolite levels under the indicated conditions (metabolomics data).

Supplemental Figure 9

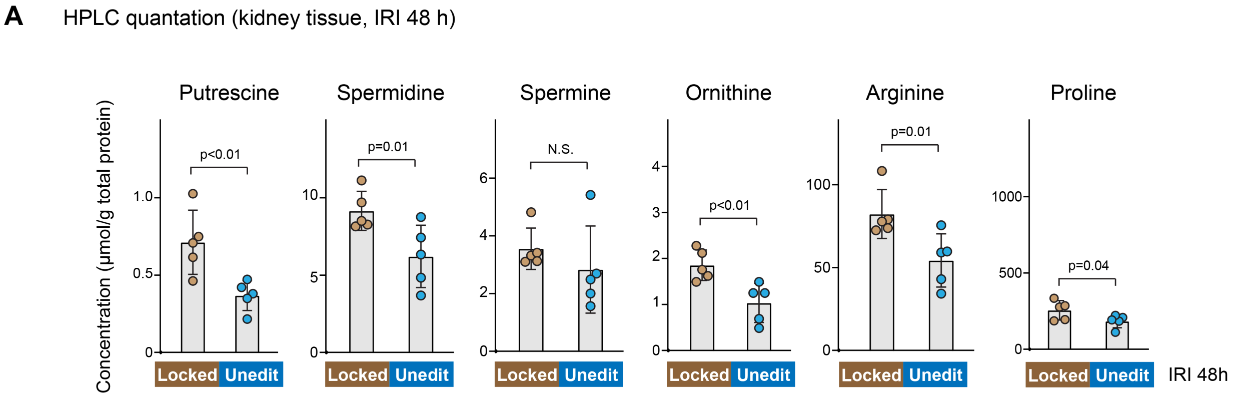

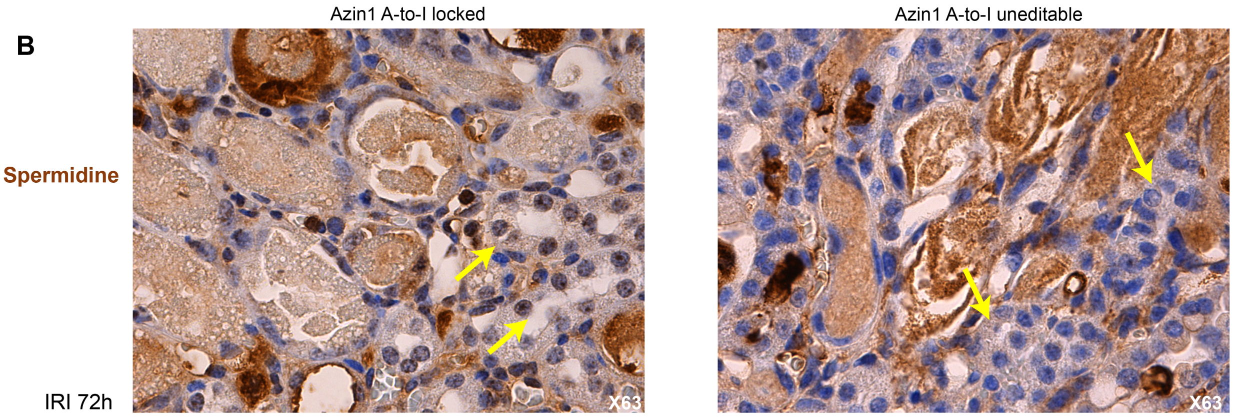

Supplemental Figure 9
(**A**) HPLC measurements of polyamines and upstream metabolites in kidney tissues 48 hours after ischemia-reperfusion injury. (**B**) Tissue staining for spermidine 72 hours after ischemia-reperfusion injury. Arrows point to spermidine staining in the A-to-I locked renal tubules (left panel) and the absence of such staining in the A-to-I uneditable renal tubules (right panel).

Supplemental Figure 10

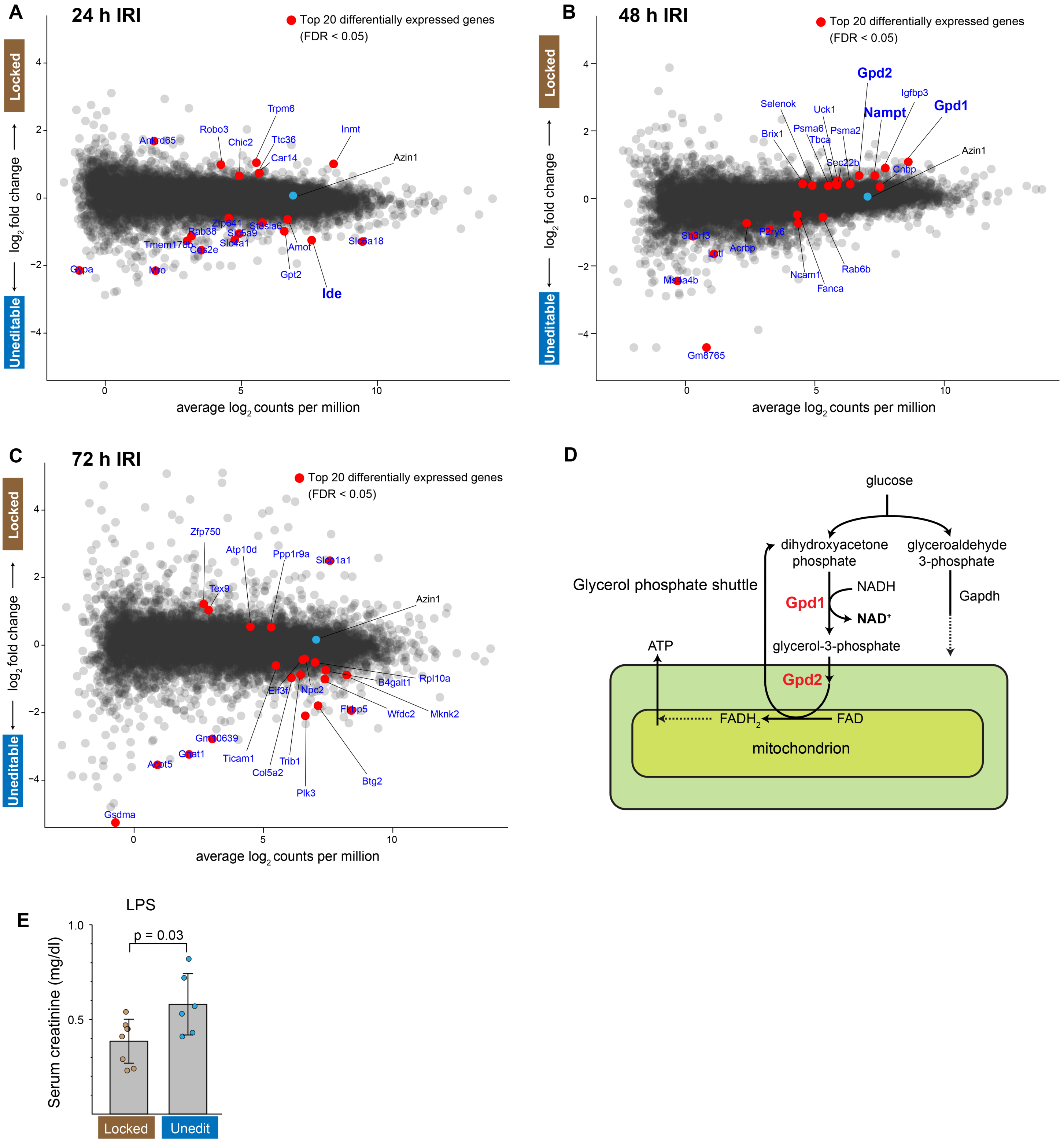

Supplemental Figure 10
(**A**-**C**) RNA-seq gene expression analysis (smear plot) comparing homozygous Azin1 A-to-I locked and uneditable mouse kidneys 24, 48, and 72 hours after ischemia-reperfusion injury. Top 20 differentially expressed genes are annotated (all FDR < 0.05). (**D**) Schematic of glycerol phosphate shuttle. (**E**) Serum creatinine levels 24 hours after 4 mg/kg LPS intravenously for homozygous Azin1 A-to-I locked and uneditable mice.

Supplemental Figure 11

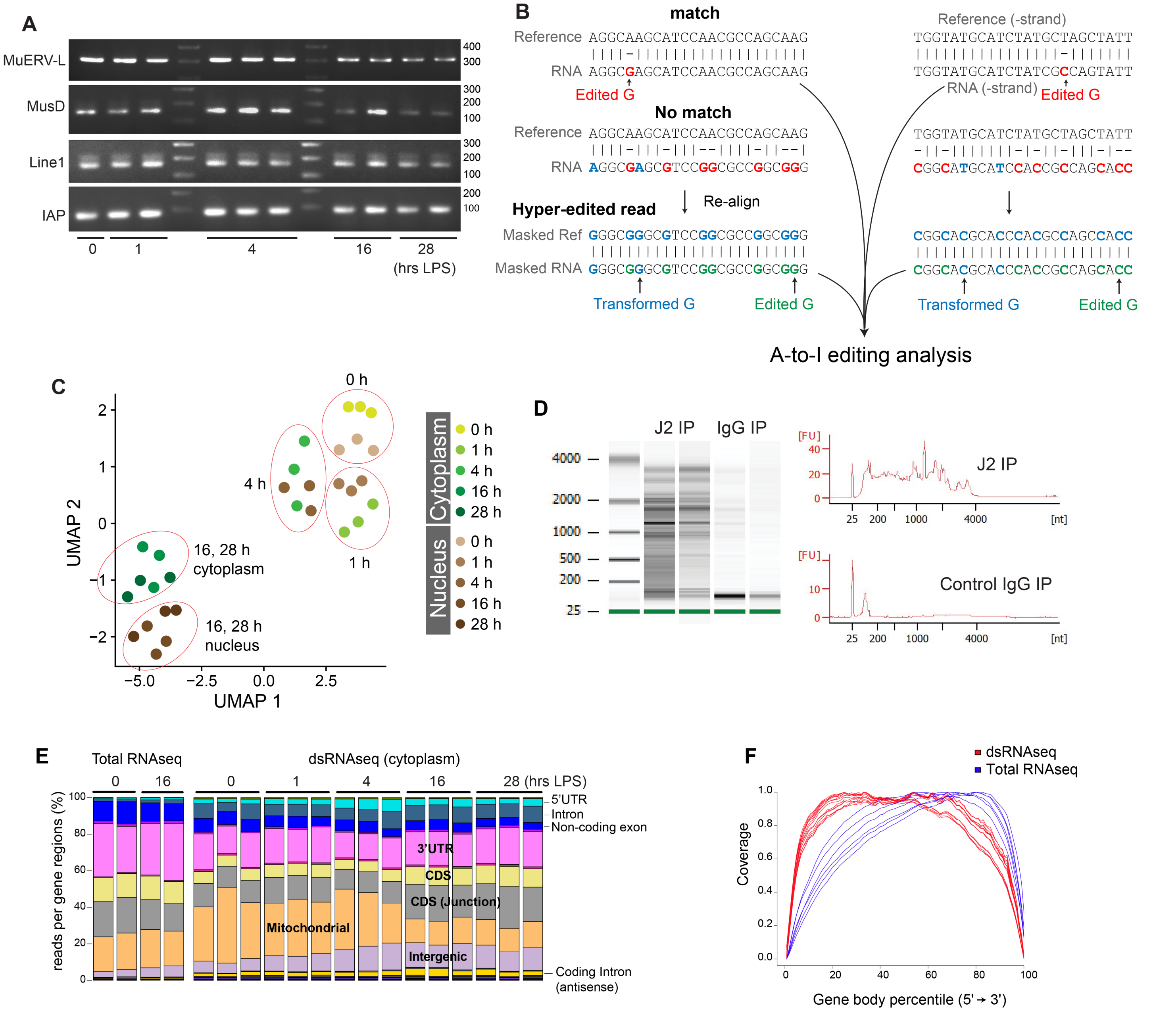

Supplemental Figure 11
(**A**) PCR gel electrophoresis of select retrotransposons in the kidney under the indicated conditions. Endogenous retroviral elements: Murine endogenous retrovirus-L (MuERV-L), MusD, and Intracisternal A particle (IAP). Long-interspersed nucleotide elements (LINEs): Line1. (**B**) Hyper-editing site alignment strategy, adopted from Porath et al (PMID: 25158696), is shown. Hyper-editing sites were identified by converting all As to Gs in both the genome reference and initially unmapped RNA reads. For clarity, stranded paired-end reads mapped to the minus strand reference is shown as T-to-C mismatches. (**C**) Distribution of dsRNA-seq samples in the UMAP space. (**D**) Representative reads length distribution after J2 dsRNA immunoprecipitation and control IgG2a kappa isotype incubation (Agilent bioanalyzer).
(**E**) Distribution of sequence reads mapped to the indicated genome features. (**F**) Distribution of sequence reads mapped to the gene body. The skewed distribution of reads toward the end of gene body in total RNAseq is due to the following:
1. the average length of the last exon is longer than that of the others and 2. there are minimal intronic reads.

Supplemental Figure 12

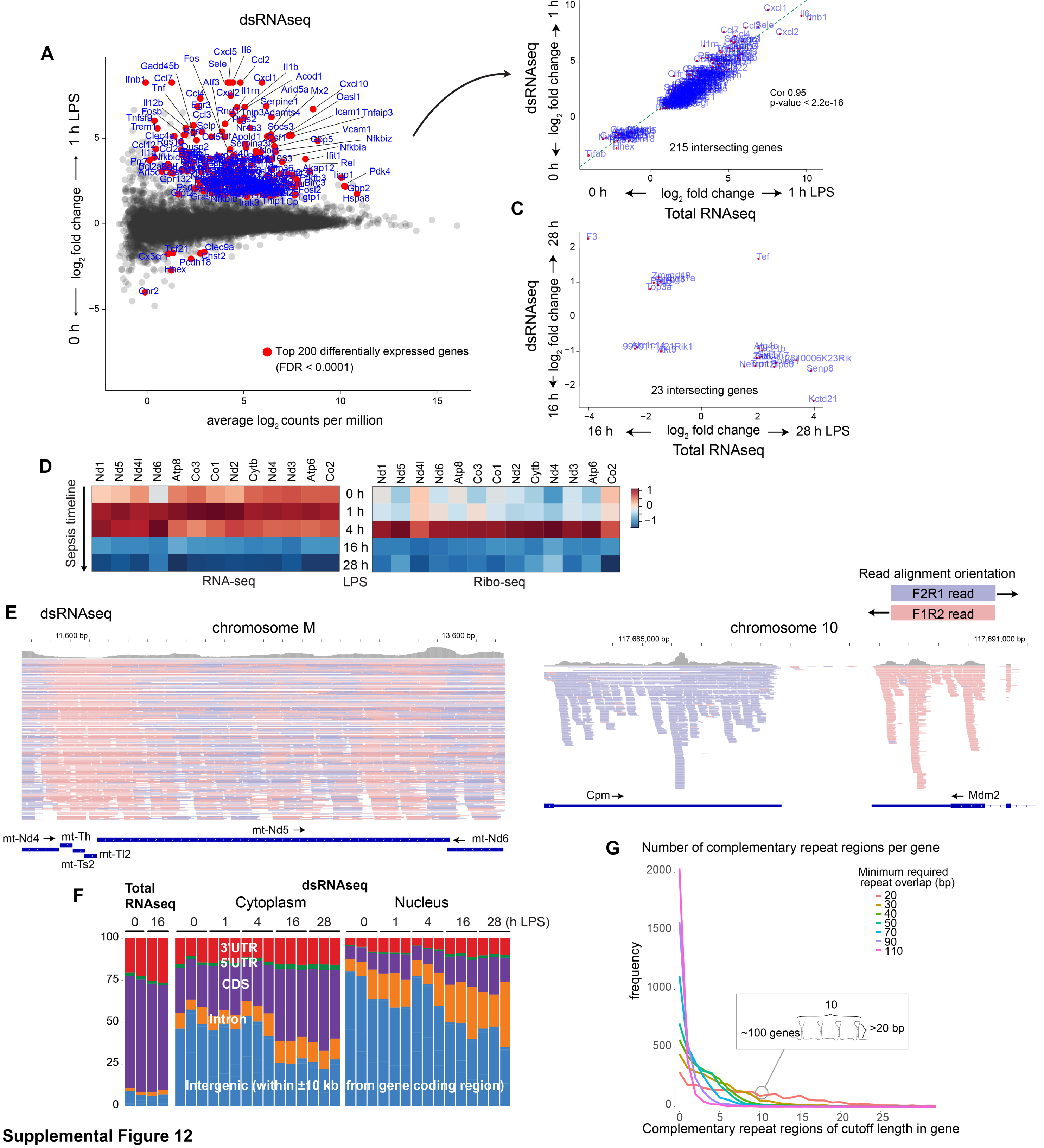

Supplemental Figure 12
(**A**) Smear plot highlighting the top 200 differentially enriched genes in the dsRNA-seq data between 1 hour after endotoxin challenge and 0-hour control. (**B**-**C**) Correlation analysis between dsRNA-seq and total RNA-seq datasets. The top 400 differentially expressed genes from each dataset under the indicated conditions were filtered for overlapping genes and plotted. (**D**) Heatmap displaying transcription and translation levels of mitochondrially-encoded mitochondrial genes over the course of endotoxemia in the kidney. (Reanalysis of published data GEO GSE120877) (**E**) Examples of dsRNA-seq read coverage distribution for the mitochondrial chromosome (left) and non-mitochondrial chromosome (right). Reads are color-coded based on the read alignment orientation (F2R1 and F1R2). The mixture of colors in the mitochondrial chromosome indicates bidirectional transcription. (**F**) Distribution of sequence reads mapped to the gene coding regions and within ±10 kb from transcription start and end sites. (**G**) Histogram summarizing the frequency of mouse genes harboring complementary repeat regions, dissected based on the lengths of palindromic sequences. This computational search was performed on the reference genome, not on our sequenced files. The x-axis denotes the number of complementary repeat regions within a given gene. The y-axis denotes the number of genes harboring complementary repeat regions.

Supplemental Figure 13

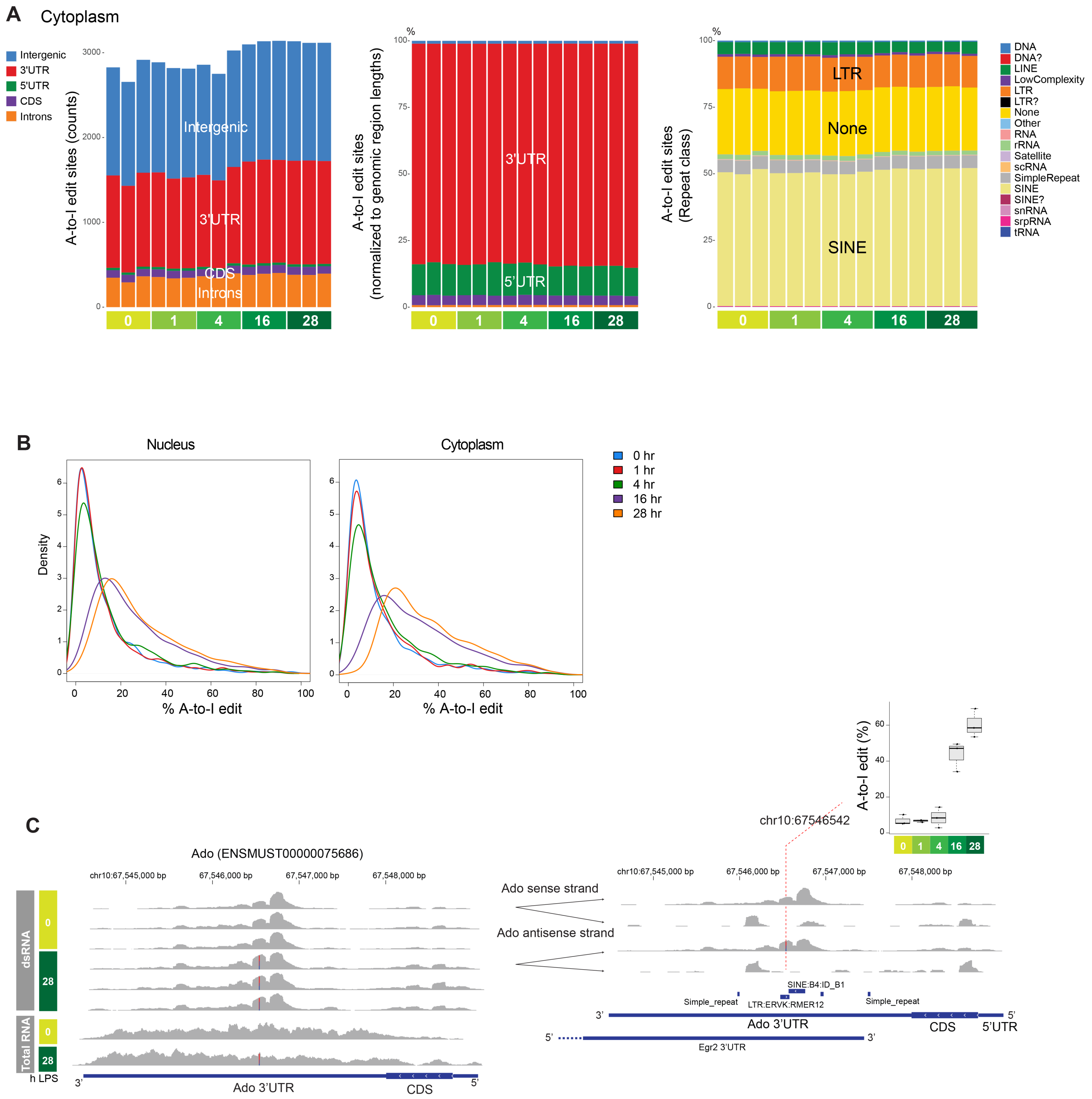

Supplemental Figure 13
(**A**) Left: Total counts and distribution of A-to-I editing sites per sample (cytoplasmic fraction; editing rate > 10% and reads count > 5 in at least 3 samples; refer to Methods for further preprocessing criteria). Middle: Distribution of A-to-I editing sites, normalized to genomic region lengths. Right: Distribution of A-to-I editing sites per repeat class.
(**B**) Density plot displaying A-to-I edit percentages, split by time points and compartments. (**C**) Example of read coverage for the gene Ado with dsRNA enrichment (top 6 tracks) and without dsRNA enrichment (bottom 2 tracks, regular total RNA sequencing). Since the Ado 3'UTR (- strand gene) overlaps with the Egr2 3'UTR (+ strand gene), reads are split into sense and antisense strands to clarify the directionality of A-to-I editing in the right panel. The upper right inset shows A-to-I editing rates at each time point for dsRNA sequencing.

Supplemental Figure 14

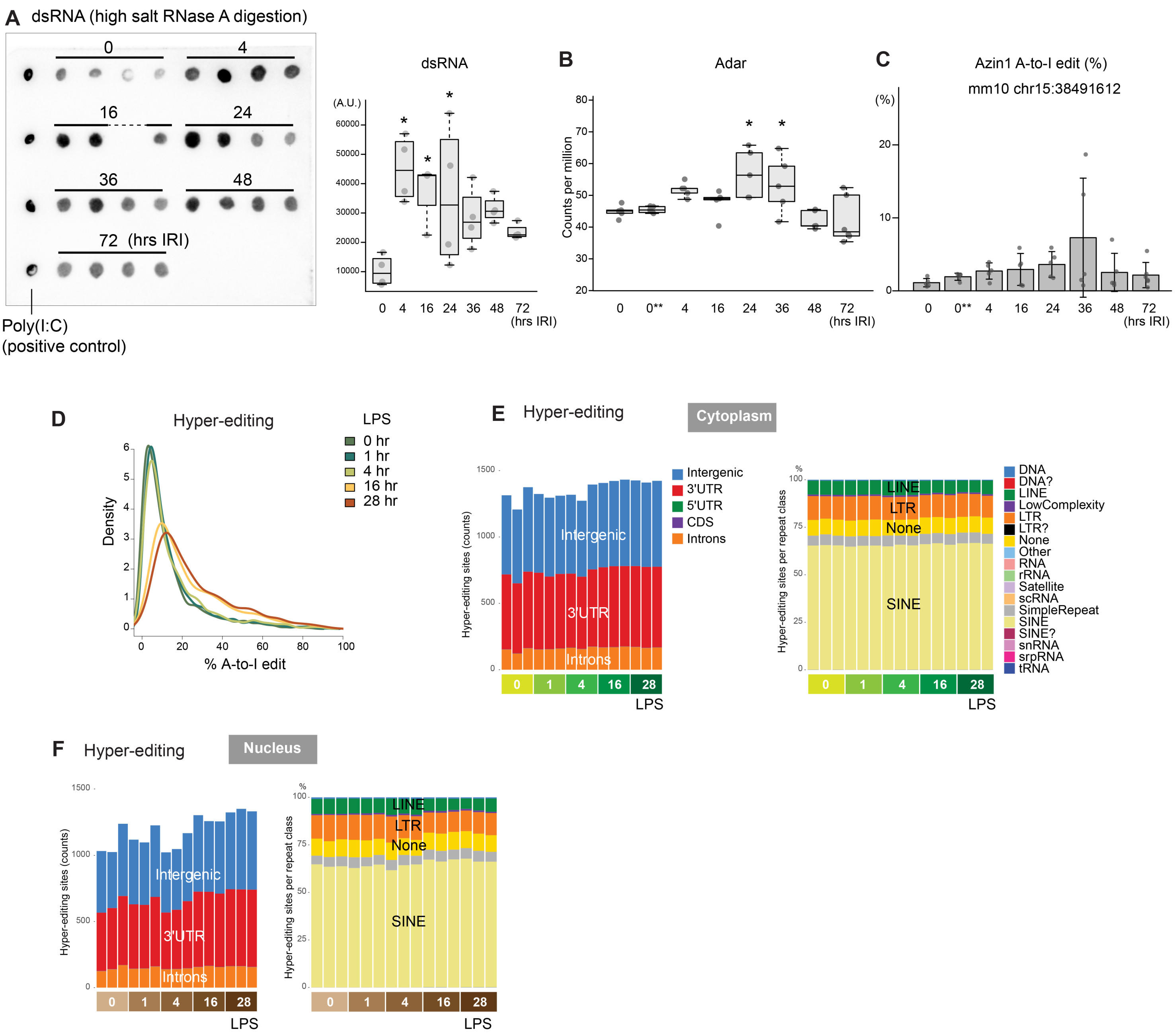

Supplemental Figure 14
(**A**) Immunoblotting of dsRNA extracted from mouse kidney tissues after ischemia-reperfusion injury at indicated time points. Single-stranded RNA was digested using RNase A with 5M NaCl. Poly(I:C) was UV crosslinked onto Hybond N+ membrane without RNase digestion. *p < 0.05 vs 0-hour control samples, 1-way ANOVA followed by Dunnett's test for multiple treatment comparisons. (**B**) Quantitation of kidney tissue Adar expression levels as determined by RNA-seq (counts per million). 0** indicates kidney tissues harvested 20 minutes after ischemia without reperfusion. *FDR < 0.05 vs 0-hour control (glmQLFTest). (**C**) Percentage of Azin1 A-to-I editing at indicated time points. (**D**) Density plot displaying A-to-I edit percentages of hyper-editing sites, split by time points and compartments. (**E**, **F**) Summary of hyper-editing sites (cytoplasm and nucleus).

Supplemental Figure 15

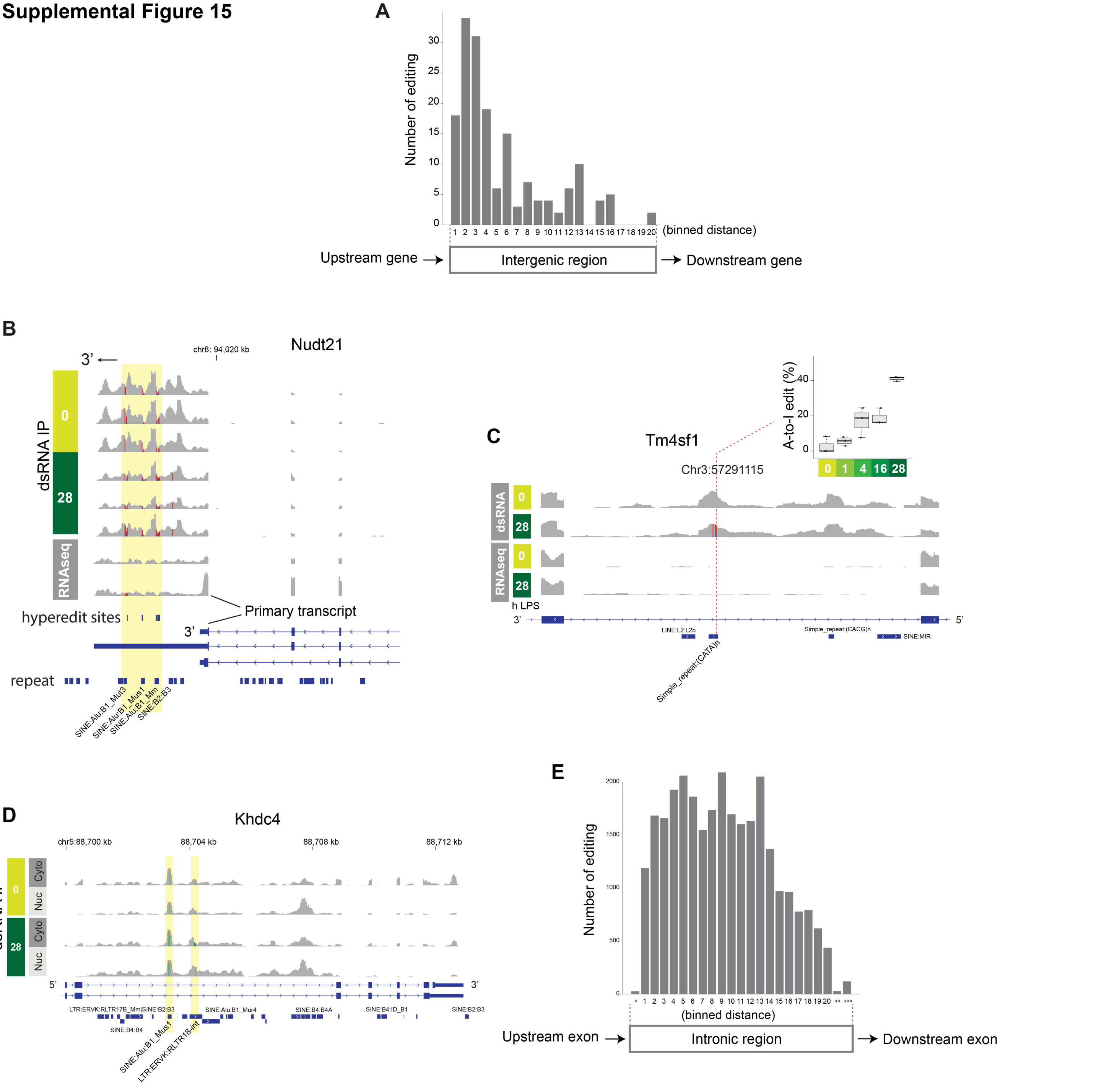

Supplemental Figure 15

(**A**) Distribution of A-to-I editing between gene coding regions. For clarity, regions flanked by genes in the same orientation are shown.
(**B**) Example of the enrichment of A-to-I edited reads after the transcription termination site of a primary transcript isoform. The top 6 tracks are dsRNA IP samples, and the bottom 2 tracks are regular total RNA sequencing (0 and 28 hours post LPS).
(**C**) Example of read coverage comparison between dsRNA enrichment (top 2 tracks) and without dsRNA enrichment (bottom 2 tracks, regular total RNA sequencing). The inset shows A-to-I editing rates at each time point after endotoxin for dsRNA sequencing. dsRNA immunoprecipitation enriched intronic reads of Tm4sf1, a tetraspanin gene, primarily in the repeat regions, both at 0-hour baseline and later time points. Significant A-to-I editing was observed only at later time points in the endotoxemia model.
(**D**) Example of read coverage comparison between nuclear and cytoplasmic fractions. Khdc4, an RNA-binding protein involved in splicing, has several A-to-I editing sites in the intronic region, corresponding to repeat regions. dsRNA immunoprecipitation enriched repeat regions in both nuclear and cytoplasmic fractions, but to a greater extent in the nuclear fraction. (**E**) Distribution of A-to-I editing sites within intronic regions. This intron analysis was done based on sites called by REDItools without further filtration steps, due to limited read coverage depth. *Splice donor site (+6 nucleotides from a splice donor site), **branch point (-40 to -10 nucleotides from a splice acceptor site). ***Splice acceptor site (-10 nucleotides from a splice acceptor site).

Supplemental Figure 16

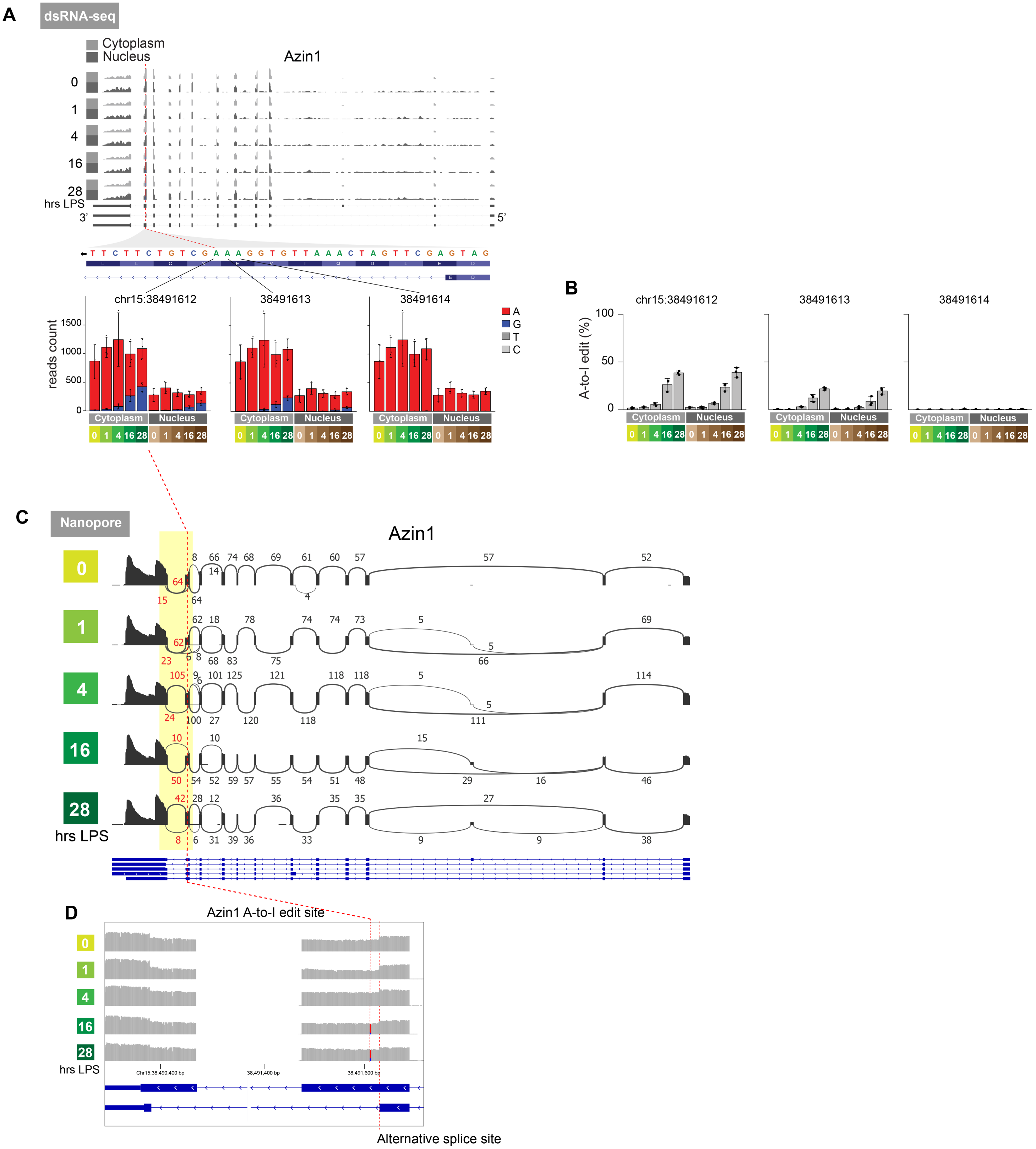

Supplemental Figure 16
(**A**) Azin1 reads distribution and A-to-I editing under the indicated conditions (dsRNA-seq data). (**B**) Percentage of Azin1 A-to-I editing. (**C**) Sashimi plot displaying reads connectivity for Azin1 under the indicated conditions (Nanopore long-read cDNA sequencing). Approximately 20% of reads near the Azin1 A-to-I editing site exhibit alternative splicing, irrespective of endotoxin time points. (**D**) Magnified view of read coverage near the Azin1 A-to-I editing site and alternative splice site.
