# Supplementary material for: Inflammation primes the murine kidney for recovery by activating AZIN1 adenosine-to-inosine editing
Source: J Clin Invest. 2024 Sep 3;134(17):e180117. doi: 10.1172/JCI180117 (PMC11364396; doi:10.1172/JCI180117)

Uncut gels

Fig 3B

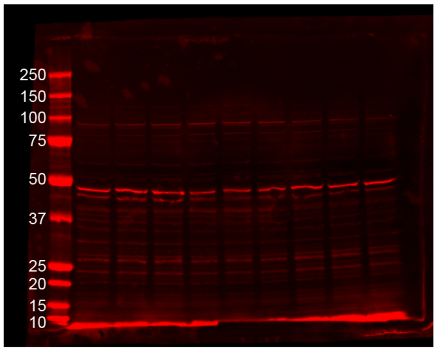

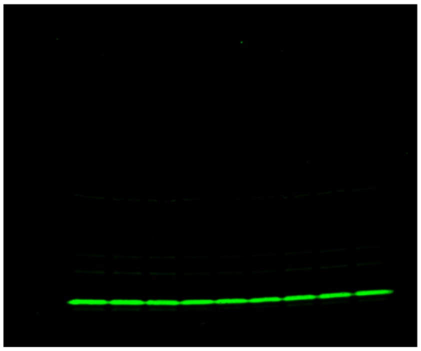

Fig 3C

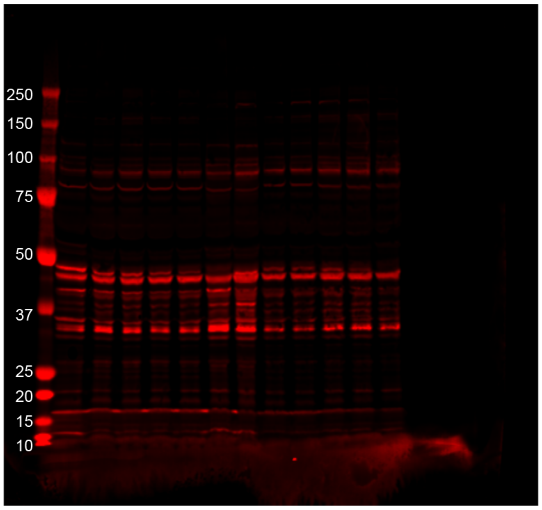

Fig 3I

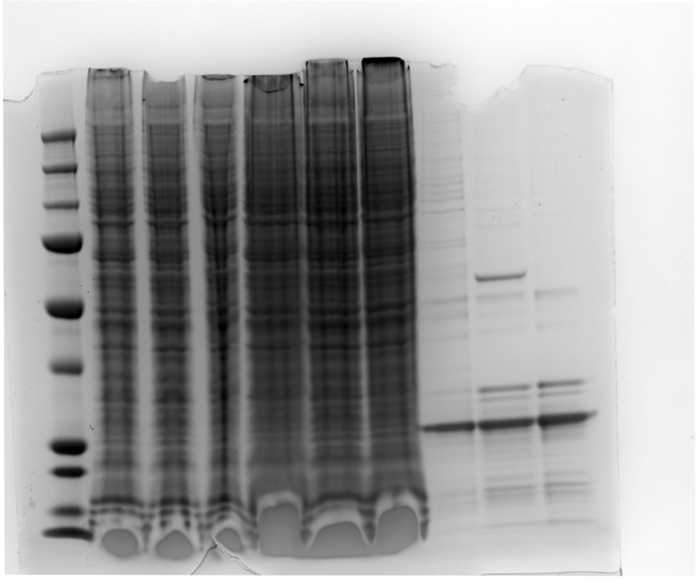

Fig 3I

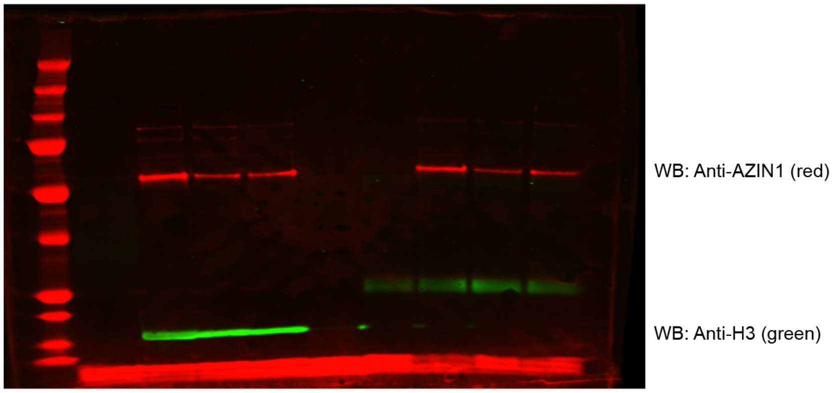

Fig 4F

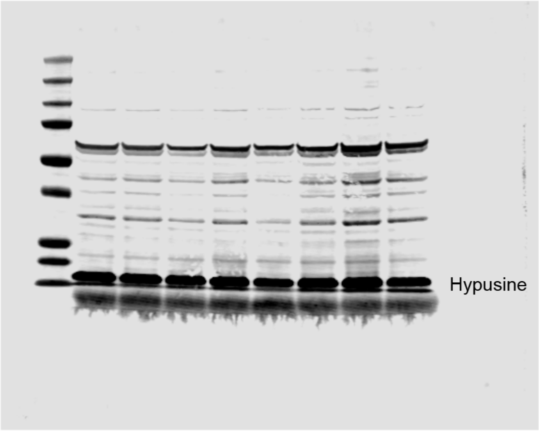

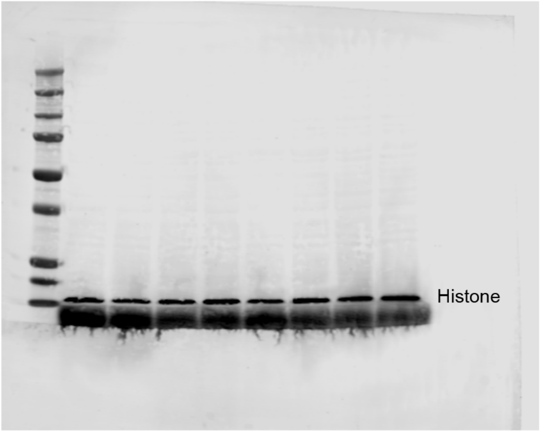

Fig 6A

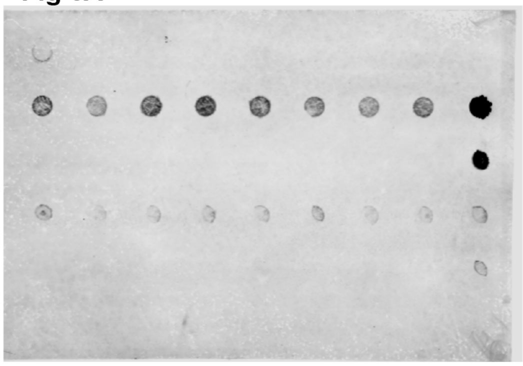

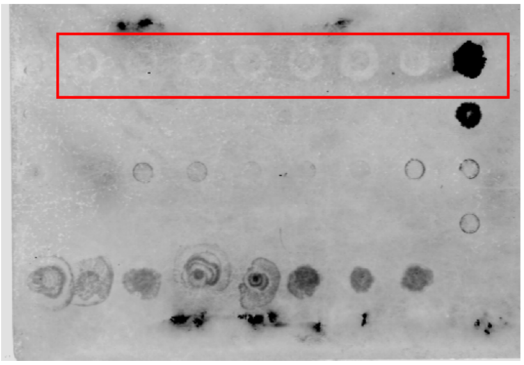

Uncut gels

Suppl Fig 5H

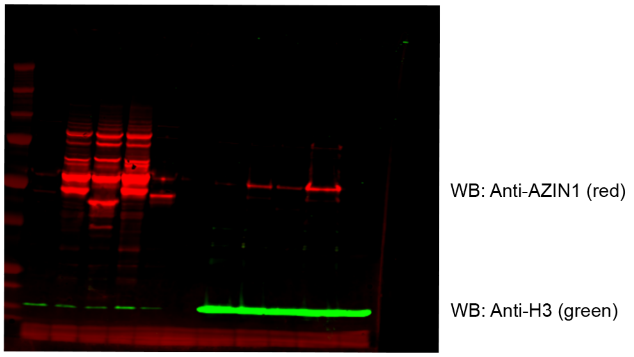

Suppl Fig 8B

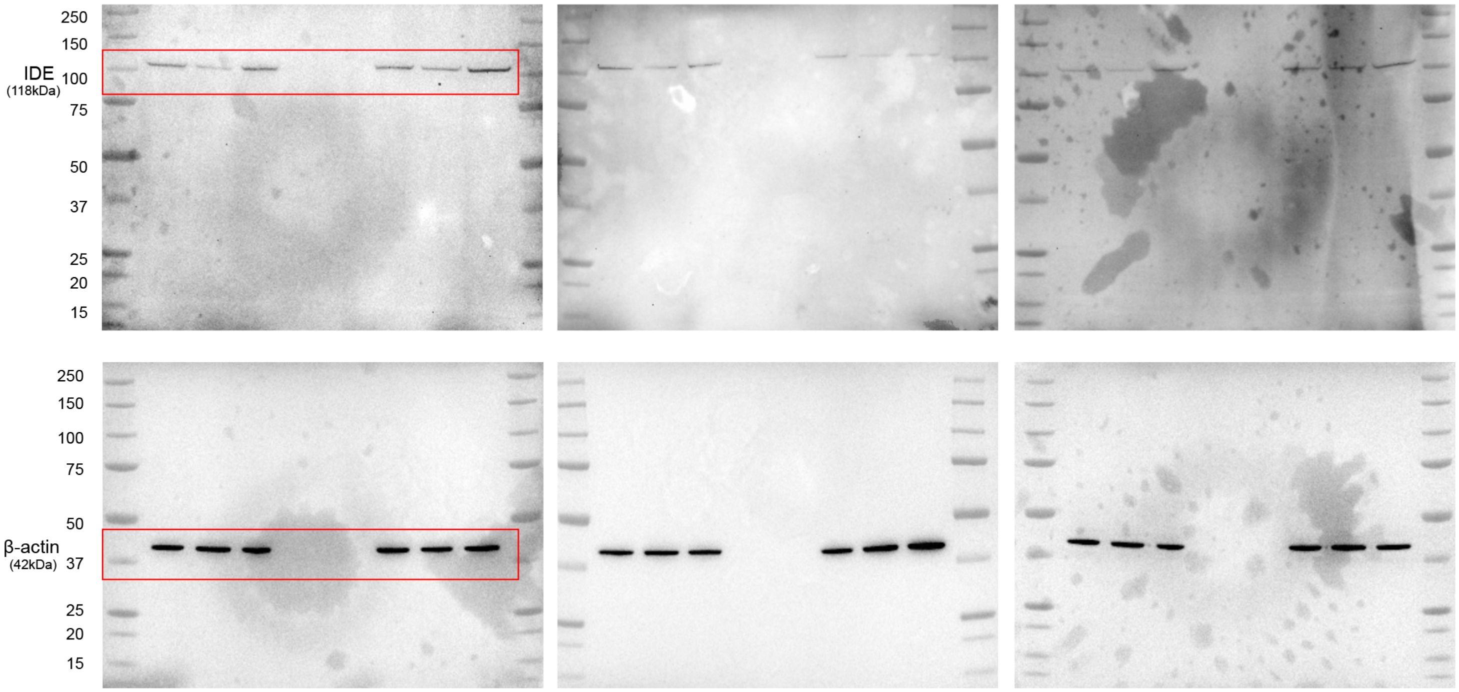

Uncut gels

Suppl Fig 11A

MuERV-L

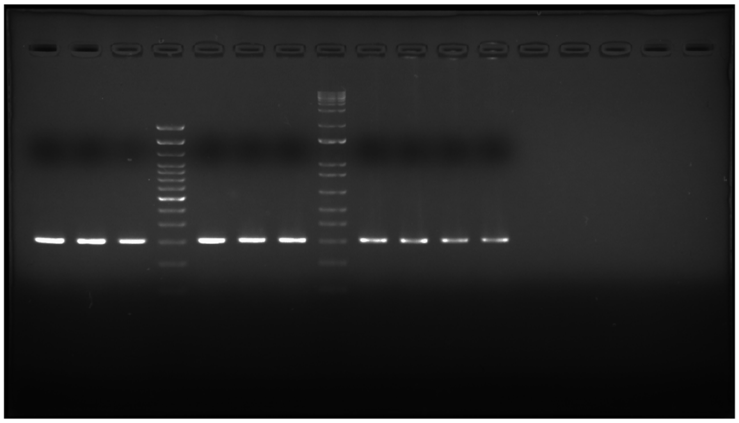

MusD

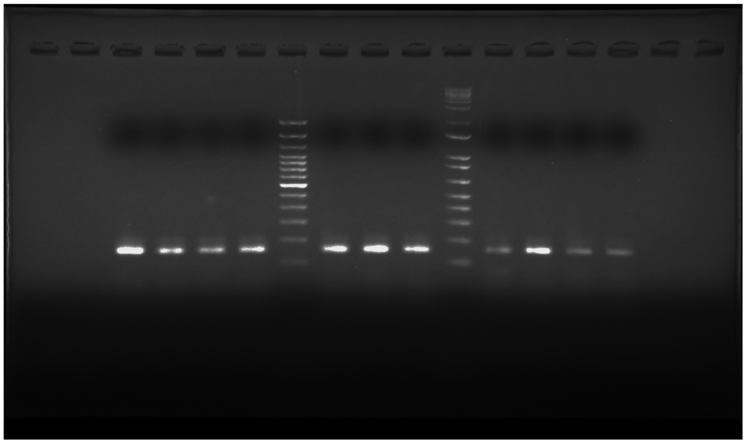

Line1

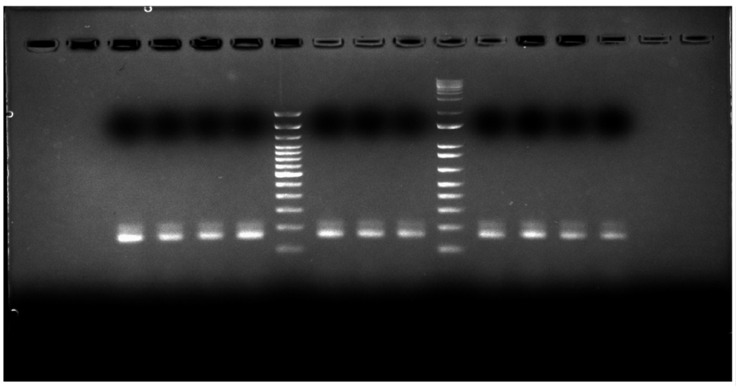

IAP

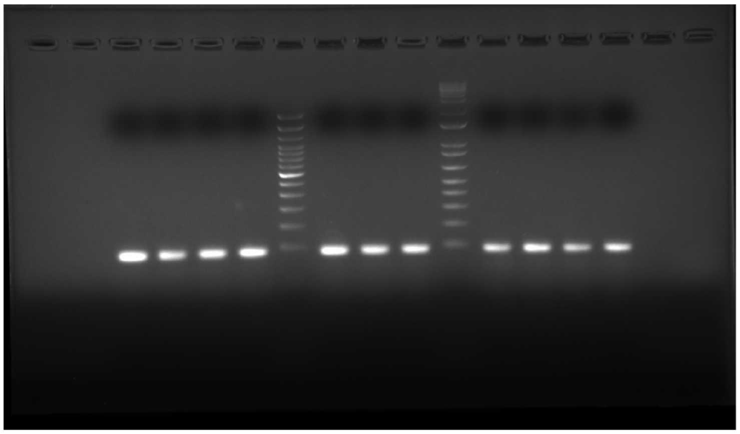

Suppl Fig 14A

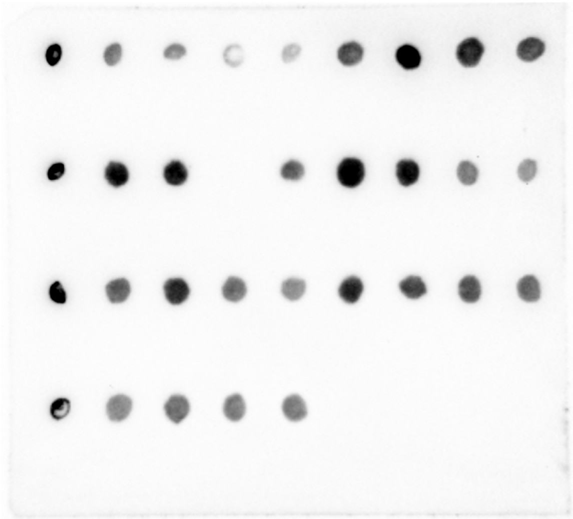

Supplement: Unedited blot and gel images [file jci-134-180117-s119.pdf]
